# What are the optimal transcranial direct current stimulation parameters and design elements to modulate corticospinal excitability? A systematic review and longitudinal meta-analysis

**DOI:** 10.1186/s42466-025-00449-1

**Published:** 2025-11-11

**Authors:** Liam C. Tapsell, Matheus D. Pinto, Ann-Maree Vallence, Casey Whife, Maria Luciana Perez Armendariz, Shaswat Senger, Jack Andringa-Bate, Dana Hince, Myles C. Murphy

**Affiliations:** 1 Nutrition and Health Innovation Research Institute, School of Medical and Health Sciences, Edith Cowan University, 270 Joondalup Drive, Joondalup, WA 6027 Australia; 2 School of Psychology, College of Health and Education, Murdoch University, Murdoch, WA Australia; 3 Centre for Healthy Ageing, Health Futures Institute, Murdoch University, Murdoch, WA Australia; 4 Medical Department, West Coast Eagles Football Club, Lathlain, WA Australia; 5 Medical School, University of Western Australia, Crawley, WA Australia; 6 La Trobe Sport and Exercise Medicine Research Centre, La Trobe University, Bundoora, VIC Australia; 7 Institute for Health Research, The University of Notre Dame Australia, Fremantle, WA Australia

**Keywords:** tDCS, Electrical brain stimulation, Stimulation parameters, Motor cortex, Cortical excitability, Facilitation, Inhibition, Neural plasticity

## Abstract

**Background:**

Corticospinal excitability, measured by motor-evoked potentials (MEPs), is often impaired in neurological and musculoskeletal conditions. Transcranial direct current stimulation (tDCS) can modulate cortical excitability and improve clinical outcomes, yet inconsistencies in parameter settings complicate identification of optimal protocols.

**Objective:**

Our primary objective was to examine the effects of: (i) stimulation polarity, (ii) duration, (iii) intensity, (iv) frequency, (v) electrode montage, and (vi) electrode design (size/shape) on MEP size.

**Methods:**

Nine databases were searched from inception to 24th November 2023. We identified 84 individual cohorts (1,709 participants) and assessed time-dependent effects of each parameter on M1 MEP-to-baseline ratio in healthy and clinical populations using multi-level longitudinal meta-analysis.

**Results:**

Anodal tDCS increased MEP size, with effects lasting up to 120 min post-stimulation. Consistent effects were observed with anodal tDCS durations ≥ 20 min and intensities ≥ 1.5 mA. Despite cohorts being matched, cathodal tDCS reduced MEP size for approximately 15 min post-stimulation, with significant effects at durations ≥ 9 min, intensity effects were inconclusive. Electrode montage and electrode size/shape influenced MEP, with greatest effect for electrodes over both the primary motor cortex and the dorsolateral pre-frontal cortex or over the cerebellar region, using 4 cm^2^ ring and 35 cm^2^ rectangular electrodes.

**Conclusion:**

tDCS effects on corticospinal excitability are parameter dependent. Anodal tDCS tends to facilitate excitability, whereas cathodal tDCS tends to inhibit excitability (depending on stimulation parameters). Specific durations, intensities, electrode placements and designs will ensure effectiveness and optimise safety. Findings support a parameter-specific approach to guide tailored neuromodulation interventions to enhance motor cortex rehabilitation outcomes.

**Supplementary Information:**

The online version contains supplementary material available at 10.1186/s42466-025-00449-1.

## Background

Neurological (e.g., stroke) and musculoskeletal (e.g., chronic lower back pain or osteoarthritis) disorders, represent a substantial health burden and socioeconomic expenditure [1, 2]. One common issue in many of these conditions is altered function of the primary motor cortex, which plays a crucial role in controlling muscle activation, learning and movement [3]. Specifically, the integrity of the motor pathway, between the primary motor cortex, corticospinal tract and muscle, is vital for performing everyday tasks and is impaired in many neurological [4] and musculoskeletal [5–8] disorders.

One way to assess this pathway and its excitability (the drive necessary to send signals to the muscle) is to elicit a motor-evoked potential (MEP) via transcranial magnetic stimulation (TMS) over the brain [9]. A larger peak-to-peak size of the MEP suggests greater excitability in the corticospinal pathway to the muscle. Excitability is often assessed through motor thresholds, which represent the minimum stimulus intensity required to evoke a muscle response. Lower motor thresholds indicate higher excitability, as less stimulation is needed to activate the pathway. Resting motor thresholds (RMT) and active motor threshold (AMT) are widely used to measure excitability during relaxed and slightly active muscle during TMS-evoked MEP measurement [10]. While the cause of variability in measures of excitability in patients with musculoskeletal disorders remain uncertain [4], any alterations could be related to the pain and disability experienced.

There are numerous influences on net excitability and the resultant MEP. Prior commentary has detailed the influence of the neuronal state, age and pathoanatomical factors of the individual [11, 12]. Within the primary motor cortex (M1) there exists distinct pathways of cortical facilitation (increasing excitability) and cortical inhibition (decreasing excitability) [5]. Short-interval intracortical inhibition (SICI) and long-interval intracortical inhibition (LICI) are two physiologically distinct mechanisms that decrease the excitability of corticospinal neurons. It is believed that, due to maladaptive neural plasticity, people with chronic musculoskeletal pain have higher than normal cortical inhibition, resulting in impaired muscle activation [4]. Therefore, people with pain are unable to produce maximal muscle force, as the brain is actively inhibiting muscle activation [13]. Cortical inhibition likely limits outcomes from exercise rehabilitation, preventing the improvement of strength/control and contributing to persistent pain and weakness [13]. In addition to inhibition, research has also reported changes in intracortical facilitation and short-interval intracortical facilitation, which all contribute to motor output [14–16].

One promising intervention shown to modulate the MEP size (corticospinal excitability) is transcranial direct current stimulation (tDCS). tDCS is a form of non-invasive brain stimulation whereby an electrical current is applied to the scalp between anode and cathode electrodes [17]. The electrical current induces a change in the underlying neurons, modulating discharge and strengthening neural connections in anodal tDCS [17]. tDCS is not a new technology; it has been extensively studied in research settings and shown to be safe over thousands of hours of application [18].

Research indicates that tDCS application can improve motor learning (skill acquisition), reaction time, muscle strength, muscle endurance, and balance, as well as affect corticospinal excitability in both healthy and clinical populations [19–23]. However, despite these positive findings, there are numerous discrepancies regarding the wide range and combination of variables that can be manipulated when prescribing tDCS [24]. These can involve the electrodes themselves (i.e., size and shape), stimulation location (e.g., pre-frontal cortex or cerebellum), or stimulation parameters (i.e., polarity, intensity, and duration) [19–21]. Therefore, it is difficult for both researchers and clinicians to determine the optimal parameters and electrode configurations to maximise changes in the MEP size. Further, the more focal the stimulation, the larger the intra-individual variability [25]. To optimise the efficacy of tDCS, to increase or decrease excitability, in both healthy and clinical populations (which is becoming increasingly common), there is a need to understand those parameters that optimise corticospinal excitability (i.e., MEP size).

Thus, the aim of this review is to assess the effectiveness of tDCS stimulation parameters, electrode locations and electrode design to determine those variables that optimally modulate excitability via MEP size. Our primary objective was to examine the effects of: (i) stimulation polarity, (ii) duration, (iii) intensity, (iv) frequency, (v) electrode montage, and (vi) electrode shape (size or shape) on MEP-to-baseline ratio.

## Methods

This systematic review was prospectively registered via PROSPERO (CRD42023486240) and reported in accordance with the Preferred Reporting Items for Systematic Reviews and Meta-analyses [26].

### Inclusion criteria

#### Participants

We included humans aged 18 years and over. We did not restrict participants based on being healthy or pathological cases. Further, we did not restrict inclusion based on any demographic features other than age.

#### Outcomes

The primary outcomes for this review were measures of motor cortex excitability. We included studies that reported any measure inclusive of: corticomedullary evoked potentials (CMEP); intracortical facilitation (ICF), including short interval ICF; motor evoked potential (MEP); motor threshold (MT); resting motor threshold (RMT); active motor threshold (AMT); short-interval intracortical inhibition (SICI); long-interval intracortical inhibition (LICI); silent period (SP). However, during data screening and extraction, it became evident that most studies primarily reported MEPs, with few studies addressing the other measures. Consequently, our primary variable of interest was the change in MEP amplitude expressed as a ratio of post-tDCS MEP amplitude to pre-tDCS MEP amplitude (known as ‘MEP-to-baseline ratio’). MEP-to-baseline ratio was our primary variable of interest as it minimises the influence of TMS intensity on MEP amplitude and allows for between study comparisons.

#### Interventions

We included any tDCS intervention study comparing tDCS stimulation parameters, electrode locations and electrode design. This included:

- Stimulation polarity (anodal versus cathodal),
- Stimulation duration (e.g., minutes),
- Stimulation intensity (e.g., milliamperes, mA),
- Stimulation frequency (e.g., sessions per day and/ or week),
- Electrode montage (e.g., electrode placement), and
- Electrode design (e.g., size or shape).

All studies were required to have a *within-study comparison* for inclusion. Further, to be included in the analysis of stimulation polarity studies were required to include matched anodal-tDCS and cathodal-tDCS samples (e.g., identical methods and matched parameters for anodal versus cathodal tDCS).

#### Types of studies

Cross-sectional, case–control and longitudinal studies (e.g., randomised controlled trials) investigating responses following tDCS using TMS were included. Studies were included regardless of their publication status (i.e., unpublished data included in clinical trial registries). Reviews were excluded. No articles required translation.

### Search strategy

Search strategies using free text terms (Appendix A) were performed from inception to 24th November 2023. Searches were performed within the following electronic databases: PUBMED, CINAHL (Full-text), EBSCO (Medline), Cochrane library, SPORTDiscus, Web of science. To reduce the risk of publication bias we also searched clinicaltrials.gov (www.clinicaltrials.gov), and the World Health Organization (WHO) International Clinical Trials Registry Platform (ICTRP) (apps.who.int/trialsearch/) for ongoing trials. Reference lists of reviews and retrieved articles were checked for additional studies missed in the electronic database search, however none were identified.

### Study selection

Identified studies were exported to reference management software, EndNote, and then uploaded into Covidence (Covidence systematic review software, Veritas Health Innovation, Melbourne, Australia). Covidence automatically removed duplicates. Two review authors (JAB and MCM) independently assessed the titles and abstracts of identified records. Articles that may have met the inclusion criteria were then assessed in full by two independent reviewers [JAB and MCM (Cohen’s Kappa for reliability = 0.86); SS and MCM (Cohen’s Kappa for reliability = 0.90)]. Disagreements were resolved by consensus.

### Assessment of methodological quality

Two review authors (LT and MLPA) independently assessed risk of bias for each study using the Joanna Briggs Institute Checklist for assessment of risk of bias for randomised controlled trials. Disagreements were resolved by consensus. This scale includes 13 items, and we defined a-priori criteria for each item (Appendix B). An overall judgement of methodological quality was assigned based on a ‘worst-item-counts’ basis with studies being assigned ‘low’ quality if at least one item was reported as “no”, unclear if no items were reported as ‘no’ and at least one item was reported as ‘unclear’, and ‘high’ quality if all items were reported as ‘yes’.

### Data extraction

The following data were extracted: study characteristics (primary author, year of publication, country of study, study design, sample size); sample characteristics (population, age, height, weight, body mass index, sex, comorbidities); stimulation parameters (polarity, stimulation target region, intensity, duration, frequency, TMS procedure, electrode size, electrode shape); TMS outcomes (CMEP, ICF, MEP, MEP-to-baseline ratio, RMT, AMT, SICI, LICI, SP). It was clear during extraction that only MEP-to-baseline had sufficient data for meta-analysis and was therefore our primary outcome variable. The other TMS variables are included in the supplementary material (Appendix C).

Data were extracted by two study authors independently (LT and MP) and all discrepancies were resolved via agreement. For manuscripts in which data were presented in figures, the outcomes of interest were estimated using a graph digitiser (WebplotDigitizer, https://automeris.io/wpd/?v=5_2). For studies in which raw MEPs were reported at each time-point, the MEP-to-baseline ratio was calculated, and the standard deviation (SD) of the ratio was estimated as follows:

Standard deviation of the ratio: MEP-to-baseline ratio × $\sqrt {\left( {\frac{SDpost}{{MEANpost}}} \right)^{2} \times \left( {\frac{SDbaseline}{{MEANbaseline}}} \right)^{2} }$.

In cases where only standard error of the mean (SEM) was reported, we calculated the SD by multiplying the SEM by the square root of the sample size. Studies that did not report any measures of dispersion had missing data handled through SD imputation from different studies with identical polarity, intensity and follow-up time. To ensure correct estimations, single-observation data points were excluded prior to analysis.

### Data synthesis

Descriptive statistics for study-level data and participant demographics are presented as counts, percentages, means, and standard deviations (or non-parametric alternatives) where applicable. The primary objective of this meta-analysis was to examine the effects of different tDCS parameters on MEP-to-baseline ratio.

For the meta-analyses, population type was categorised into ‘healthy individuals’ or ‘clinical populations’ (e.g., individuals with Parkinson’s disease, spinal cord injury, chronic low back pain, and stroke). One meta-analysis was conducted for each of the six objectives to examine the impact of the six tDCS parameters [(i) stimulation polarity, (ii) duration, (iii) intensity, (iv) frequency, (v) electrode montage, and (vi) electrode shape (size or shape)] on MEP-to-baseline ratio, with each analysis modelled using a multilevel (hierarchical) framework with restricted maximum likelihood. All analyses were conducted in R (v.4.3.3, R Core Team, https://www.r-project.org/) using RStudio environment (v. 2024.4.1.748, RStudio Team, https://www.rstudio.com/). Meta-analyses were performed using ‘metafor’ package (v. 4.6-0) [27] and the estimated marginal means used for visualisation computed using ‘emmeans’ package (v. 1.10) [28]. Visualisations were produced with ‘ggplot2’ (v3.5.1). [29]

We employed two-level hierarchical models for all meta-analyses, as most studies reported multiple MEP measurements over time for the same individuals. To account for this repeated-measures structure, random intercepts were included at the study level (~ 1|Study_ID). For the stimulation polarity analysis, however, a three-level model was applied, nesting anodal and cathodal stimulation data were nested within each study, thereby introducing an additional level of random effects (~ 1|study_id/polarity). F-tests were used to evaluate main effects and degrees of freedom are provided. Confidence intervals for all models were based on a t-distribution given the small sample size of studies included in each meta-analysis. Statistical significance was set at *p* < 0.05, and models were adjusted to include relevant moderators depending on the research question. The specifics for each meta-analysis are presented in Appendix D.

### Statistical heterogeneity

The statistical significance of residual heterogeneity among studies was examined using the Q-statistics derived from the residual heterogeneity test (Q), with a significant *p*-value indicating unexplained variability in effects after accounting for moderators, i.e., the presence of heterogeneity. The calculated Q-statistic and *p*-value were extracted to summarise residual heterogeneity in the meta-analyses.

To quantify the proportion of total variance in effects attributable to heterogeneity among studies, the overall I^2^ statistic was calculated, taking into account both the hierarchical structure of the data and the included moderators. This approach allowed for the estimation of total variance in effects due to heterogeneity among studies, while accounting for both between-cluster and within-cluster variability, particularly in analyses involving nested structures such as polarity.

### Assessment of the quality of the body of evidence

Quality of the body of evidence was assessed using the GRADE approach, which involves making an overall judgement on the quality of the body of evidence based on the overall risk of bias, consistency of results, directness of the evidence, imprecision and publication bias. Certainty was downgraded when: (i) studies were of low-quality, (ii) studies included < 200 participants [30], (iii) there was evidence of inconsistency, (iv) there was evidence of indirectness, (v) there was evidence of imprecision, and (vi) there was publication bias (funnel plots were visually inspected to explore the likelihood of reporting biases when at least 10 studies were identified) [31].

## Results

**Fig. 1:**
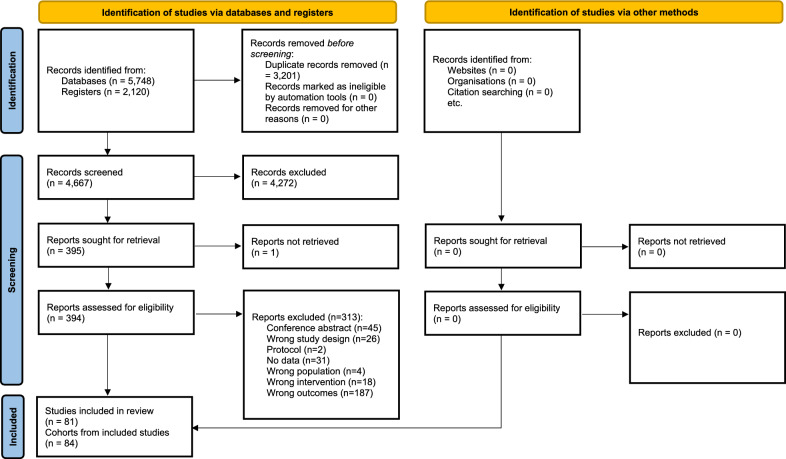
PRISMA flow chart

Our search identified 7868 records, of which 3201 duplicates were removed by Covidence and manual searching, leaving 4667 records for title and abstract screening (Appendix E). Full-text screening of 395 records resulted in the exclusion of 314 records (Appendix F). Thus, this systematic review included data from 81 records [32–112], consisting of 84 distinct cohorts (Fig. 1).

### Study characteristics

Complete study characteristics are displayed in Table 1. The 84 cohorts were predominantly from Germany (n = 25, 30%) and Australia (n = 18, 21%). A cross-over design was most commonly utilised (n = 68, 81%). Non-pathological (apparently healthy) populations made up most cohorts (n = 75, 89%), with other studies exploring populations with stroke (n = 5, 6%), Parkinson’s disease (n = 2, 2%), Spinal Cord Injury (n = 1, 1%), and Lower Back Pain (n = 1, 1%).

**Table 1:** Included study characteristics

Author	Country	Design	Population	Sample size	Female Sex (%)	Mean Age (SD)—years	Mean height (SD)—cm	Mean weight (SD)—kg	Mean BMI (SD)—kg/m [2]
Agboada [32]	Germany	Cross-over	Healthy	16	56	26.5 (2.2)			
Alonzo [33]	Australia	Cross-over	Healthy	12	0	21.4			
Ammann [34]	USA	Cross-over	Healthy	12	33	24.9 (5.1)			
Anguis [35]	UK	Cross-over	Healthy	12	33	24.0 (5.0)	175.0 (12.0)	74.0 (17.0)	24.16
Azabou [36]	France	Cross-over	Healthy	12	0	27.0 (4.0)			
Baltar [37]	Brazil	Cross-over	Healthy	12	75	23.5 (3.3)			
Bashir [38]	Saudi Arabia	Cross-over	Healthy	32	38	28.0 (10.9)			
Bashir [39]	Saudi Arabia	Cross-over	Healthy	28	36	24.1 (5.2)			
Bastani [42]	Australia	Cross-over	Healthy	12	83	21.8 (1.4)	168.6 (3.1)	62.4 (3.1)	21.95
Bastani [40,41]	Australia	Cross-over	Healthy	12	58	34.5 (10.3)	168.9 (15.5)	68.6 (11.0)	24.05
Batsikadze [43]	Germany	Parallel	Healthy	14	64	25.8 (3.7)			
Behrangrad [44]	Australia	Cross-over	Healthy	21	48	23.7 (4.5)			
Bocci [45]	Italy	Cross-over	Healthy	15	47	25.8 (5.9)			
Boros [46]	Germany	Parallel	Healthy	16	0	24.1 (2.3)			
Cabibel [47]	France	Cross-over	Healthy	17	29	27.4 (8.7)			
Chew [48]	Australia	Cross-over	Healthy	29	48	22.8 (3.1)			
Christova [49]	Austria	Parallel	Healthy	36	61	26.2 7.9)			
Cosentino [50]	Italy	Cross-over	Parkinsons Disease	14	43	58.0 (12.1)			
Faber [51]	Germany	Cross-over	Healthy	15	0	25.8 (2.4)			
Farnad [52]	Germany	Cross-over	Healthy	16	50	59.1 (4.5)			
Farnad m[52]	Germany	Cross-over	Healthy	16	50	73.1 (5.0)			
Feurrra [53]	Italy	Cross-over	Healthy	11	36				
Foerster [54]	Germany	Cross-over	Healthy	16	63	25.3 (4.3)			
Furubayashi [55]	Japan	Cross-over	Healthy	8	13				
Galea [56]	USA	Cross-over	Healthy	16	38	26.0 (7.0)			
Galvez [57]	Australia	Cross-over	Healthy	11	0	24.4 (4.1)			
Ghasemian-Shirvan [58]	Germany	Cross-over	Healthy	20	45	25.9 (3.4)			
Ghasemian-Shirvan [58]	Germany	Cross-over	Healthy	20	45	58.6 (5.1)			
Ghasemian-Shirvan [58]	Germany	Cross-over	Healthy	20	45	74.4 (4.6)			
Haeckert [59]	Germany	Cross-over	Healthy	15	67	23.9 (3.0)			
Hashemirad [60]	Australia	Parallel	Healthy	51	71	25.82 (6.14)			
Hassanzahraee [60]	Australia	Cross-over	Healthy	15	53	24.7 (7.5)			
Hill [61]	Australia	Cross-over	Healthy	20	60	29.1 (12.3)			
Horvath [62]	Australia	Cross-over	Healthy	14	50	22.5 (4.1)			
Isis [63]	Brazil	Cross-over	Healthy	15	60	25.8 (5.0)			
Jamil [64]	Germany	Parallel	Healthy	29	52	25.0 (4.4)			
Jefferson [65]	UK	Cross-over	Healthy	17	59	37.6			
Jeffrey [66]	Canada	Cross-over	Healthy	8	38	25.3 (6.0)			
Khedr [67]	Egypt	Parallel	Stroke	40	35	58.4 (8.8)			
Kidgell [68]	Australia	Cross-over	Healthy	14	43	27.5 (7.7)	162.7 (42.3)	73.0 (14.6)	27.58
Kindred [69]	USA	Cross-over	Stroke	21		64.8 (12.5)	170 (10)	185(37)*	29.5
Kuo [70]	Germany	Cohort	Healthy	14	57	25.29 (3.2)			
Kuo [71]	Taiwan	Cross-over	Stroke	14	64	63.0			
Laczo [72]	Germany	Cross-over	Healthy	10	50	27.4 (3.9)			
Lampropoulou [73]	UK	Cross-over	Healthy	12	67	32.0 (6.0)			
Lefebvre [74]	USA	Parallel	Healthy	30	0	27.0 (3.8)			
McCambridge [75]	New Zealand	Cross-over	Healthy	18	50	25.9			
Moliadze [76]	Germany	Cross-over	Healthy	12	0	27.0 (3.0)			
Monte-Silva [78]	Germany	Cross-over	Healthy	12	58	24.3 (6.1)			
Monte-Silva [77]	Germany	Cross-over	Healthy	15	60	25.5 (3.6)			
Mooney [79]	New Zealand	Cross-over	Healthy	16	56	26.0 (1.0)			
Mordillo-Mateos [80]	Spain	Cross-over	Healthy	15	0				
Mosayebi-Samani [82]	Germany	Cross-over	Healthy	18	39	26.6 (3.6)			
Murray [83]	USA	Cross-over	Spinal Cord Injury	9	44				
NCT03481309 [102]	USA	Cross-over	Healthy	19	0	24.31 (5.84)			
Nitsche [84]	Germany	Cross-over	Healthy	12	58	25.0 (4.0)			
Nitsche [85]	Germany	Cross-over	Healthy	12	75	22.5			
O'Shea [86]	UK	Cross-over	Healthy	13	62	24.6 (4.8)			
Pellerini [87]	Australia	Cross-over	Healthy	15	0	28.5 (8.3)			
Pillen [88]	Germany	Cross-over	Healthy	11	73	24.1 (2.4)			
Pillen [88]	Germany	Cross-over	Healthy	20	40	27.5 (2.4)			
Power [89]	Canada	Cross-over	Healthy	10	50	27.4 (9.2)			
Puri [90]	Australia	Cross-over	Elderly	33	64	66.0 (4.8)			
Rivera-Urbina [91]	Germany	Cross-over	Healthy	37	54	28.6 (8.0)			
Samani [81]	Germany	Cross-over	Healthy	16	56	25.1 (0.6)			
Santarnecchi [92]	Italy	Cross-over	Healthy	10	50	26.0 (3.0)			
Sasaki [93]	Japan	Pre-post	Healthy	16	25	21.6 (1.0)			
Schabrun [94]	Australia	Cross-over	Healthy	21	62	22.0 (2.0)			
Shah [95]	USA	Cross-over	Healthy	8	38				
Sohn [96]	Korea	Parallel	Healthy	28	46	22.6 (1.2)			
Strube [97]	Germany	Cross-over	Healthy	59	53	28.0 (7.7)	174.2 (94.5)	71.4 (17.7)	23.52
Suzuki [98]	Japan	Case–control	Stroke	7	14	64.5			
Thapa [99]	Australia	Case–control	Lower Back Pain	50	48	45.0 (16.0)			
Tremblay [100]	Canada	Cross-over	Healthy	40	50	24.6 (4.4)			
Uehara [101]	USA	Cross-over	Healthy	30	47	24.1 (4.3)			
Vaseghi [106]	Australia	cross-over	healthy	12	67	24.0 (2.1)			
Vaseghi [103]	Australia	Cross-over	Healthy	12	75	25.0 (1.3)			
Vaseghi [104, 105]	Australia	Cross-over	Healthy	12	67	23.6 (5.3)			
Vignaud [107]	France	Cross-over	Healthy	14	79	27.5 (4.6)			
Vitale [108]	Spain	Parallel	Healthy	50	86	19.6 (1.3)			
Wiethoff [109]	UK	Cross-over	Healthy	53	62	26.8 (9.0)			
Wiltshire [110]	UK	Parallel	Healthy	60	50	22.3 (4.8)			
Wong [111]	Taiwan	Parallel	Parkinsons Disease	36	47	54.2 (4.1)			
Wong [112]	Taiwan	Parallel	Stroke	48	21	52.7			

*BMI* Body mass index, *RCT* Case–control, *USA* United States of America, *UK* United Kingdom, * = likely miscalculation in original paper

### Participant characteristics

Complete participant characteristics are reported in Table 1. In the 84 included cohorts there was a total sample of 1709 participants, with cohorts ranging from 7 to 60 participants (median being n = 15). Participants’ sex was reported by 83 cohorts, with female sex inclusion ranging from 0 to 86% of participants (median being 50% female sex). Age was reported in 79 cohorts, with mean age ranging from 19.6 to 74.4 years. Height was reported in 6 cohorts, with mean height ranging from 162.7 to 175.0 cm. Body mass was reported in 6 cohorts, with mean body mass ranging from 62.4 to 74.0 kg. Body mass index (BMI) was reported in 6 cohorts, with mean BMI ranging from 21.9 to 29.5 kg/m^2^. Two studies reported participants had comorbidities; one study reported participants had hypertension, diabetes, and hyperlipidaemia [67] while another study reported participants had pons infarctions. [71]

### Risk of bias

The overall study quality was low with 96% of cohorts (n = 81) being rated as low quality (Appendix G). The main reasons for low quality were lack of blinding of the person delivering the intervention (n = 67, 83%), the person performing the assessment (n = 61, 75%), and the participant (n = 20, 25%).

### Stimulation polarity

**Fig. 2:**
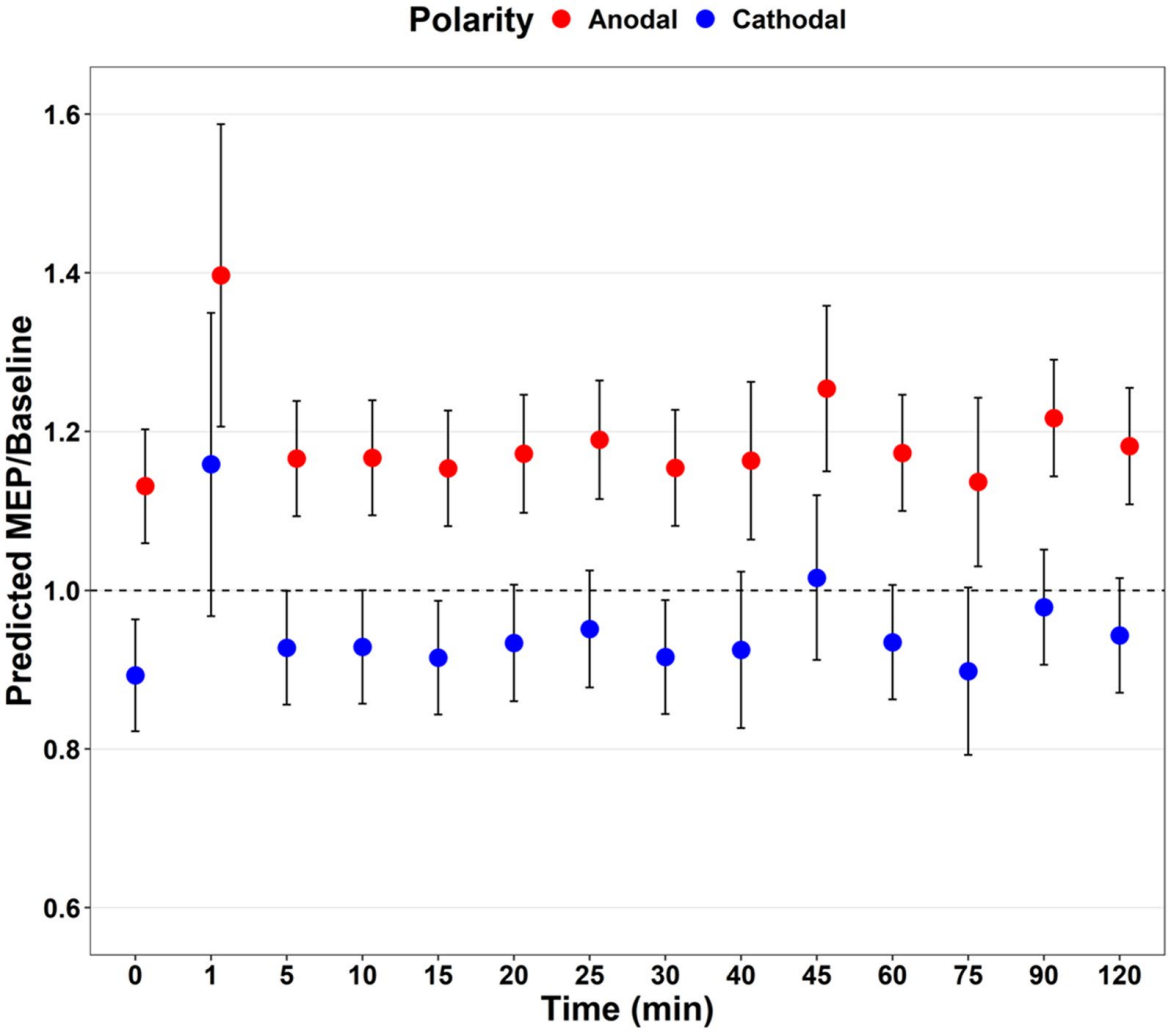
Predicted values of motor evoked potential-to-baseline over time following tDCS application: Red—Anodal tDCS, Blue—Cathodal tDCS

The temporal effects of tDCS on MEP-to-baseline ratio were evaluated from immediately post-tDCS to 120 min post-tDCS and included data from 40 studies (n = 677 participants). There was a significant main effect on the MEP at nearly all timepoints following tDCS (F_18,515_ = 17.97, *p* < 0.001, Fig. 2, Appendix H, Appendix I). Anodal polarity was associated with a larger MEP-to-baseline ratio, cathodal tDCS was associated with a smaller MEP-to-baseline ratio (*p* < 0.001). The MEP-to-baseline ratio size was negatively associated with age (*p* < 0.001), positively associated with stimulation duration (*p* < 0.001), and not associated with tDCS intensity (*p* = 0.86). Pathological cases versus health controls exhibited different response sizes in the MEP-to-baseline ratio (*p* < 0.01). Pooled study estimates are presented in Appendix I, demonstrating heterogeneity (Q = 5610.91, *p* < 0.001, I^2^ = 94.1). The certainty of the evidence was ‘very-low’ after being downgraded for risk of bias, small sample bias and inconsistency.

### Stimulation duration

#### Anodal tDCS

**Fig. 3:**
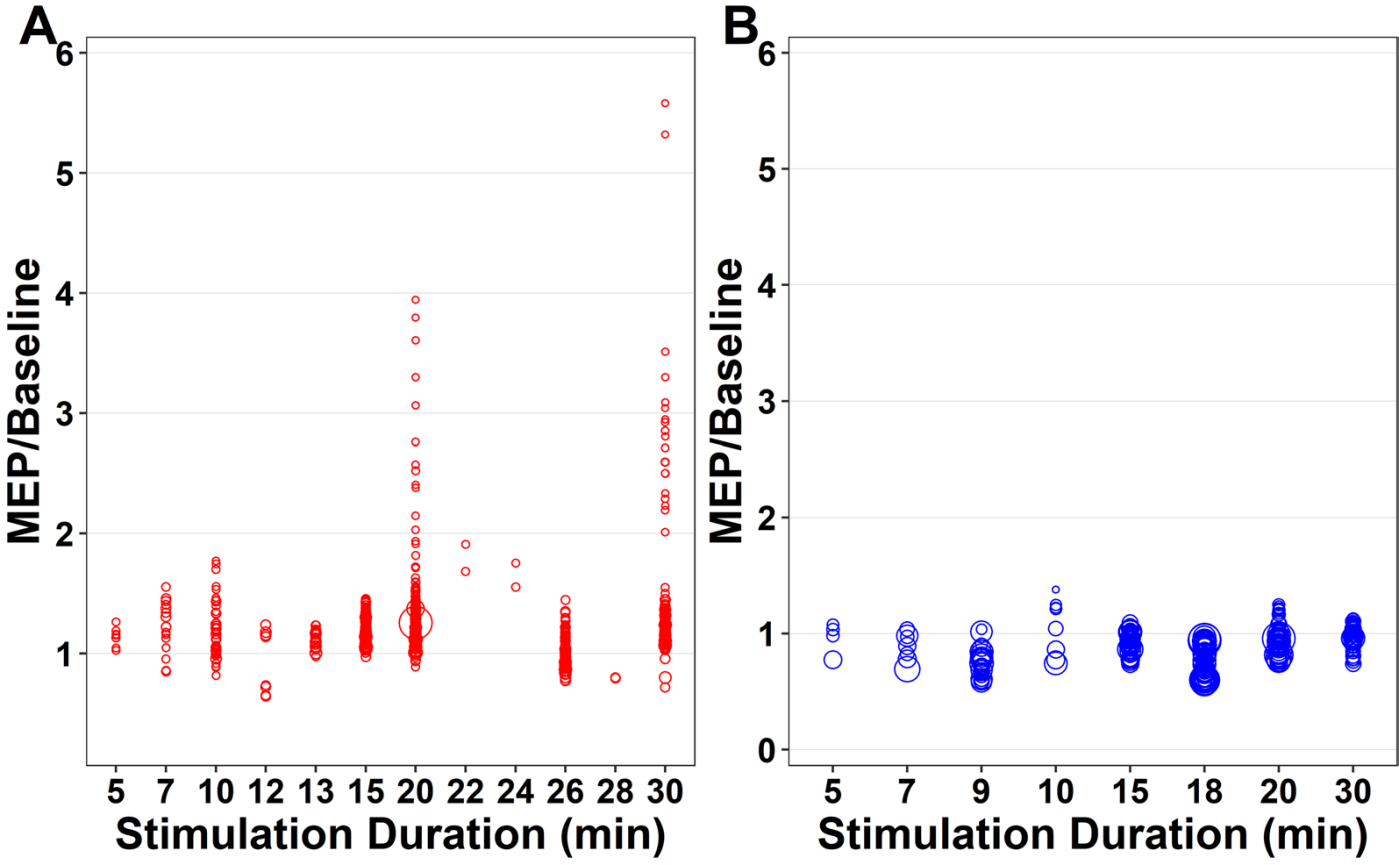
Individual study level effects of anodal and cathodal transcranial direct current stimulation over time (Stimulation Duration) (**A**) Anodal tDCS, (**B**) Cathodal tDCS

The effect of stimulation duration for anodal tDCS on MEP-to-baseline ratio was evaluated from immediately post-tDCS to 1,920 min (32 h) post-tDCS and included data from 11 studies (n = 279 participants). There was a significant main effect for stimulation duration (F_32,558_ = 21.88, *p* < 0.001, Fig. 3, Appendix J, Appendix K), with an increase in the MEP-to-baseline ratio at ≥ 20 min of stimulation. MEP-to-baseline ratio was negatively associated with age (*p* < 0.001), and positively associated with stimulation intensity (*p* < 0.001), and differences between healthy and pathological populations were observed (*p* < 0.001). Pooled study estimates are presented in Appendix K and demonstrated heterogeneity (Q = 1573.74, *p* < 0.001, I^2^ = 94.8). The certainty of the evidence was ‘very-low’ after being downgraded for risk of bias, small sample bias and inconsistency.

#### Cathodal tDCS

The effect of stimulation duration for cathodal tDCS on MEP-to-baseline ratio was evaluated from immediately post-tDCS to 1,920 min post-tDCS and included data from 5 studies (n = 82 participants). There was a significant main effect for stimulation duration (F_28,224_ = 12.92, *p* < 0.001, Fig. 3, Appendix K, Appendix L), with a decrease in the MEP-to-baseline ratio at ≥ 9 min of stimulation. MEP-to-baseline ratio was positively associated with age (p = 0.02) but not stimulation intensity (*p* = 0.930). Pooled study estimates are presented in Appendix K and demonstrated heterogeneity (Q = 847.41, *p* < 0.001, I^2^ = 45.0). The certainty of the evidence was ‘very-low’ after being downgraded for risk of bias, small sample bias and inconsistency**.**

### Stimulation intensity

#### Anodal tDCS

**Fig. 4:**
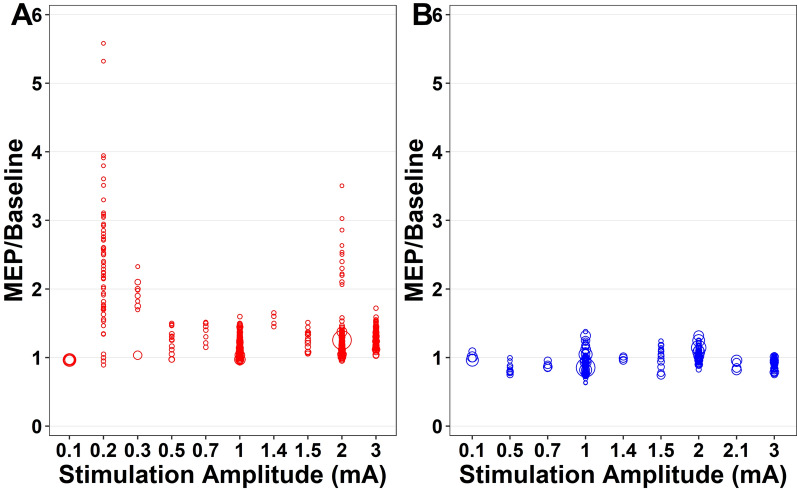
Individual study level effects of anodal and cathodal transcranial direct current stimulation over time (Stimulation Intensity): (**A**) Anodal tDCS, (**B**) Cathodal tDCS

The effect of stimulation intensity for anodal tDCS on MEP-to-baseline ratio was evaluated from immediately post-tDCS to 1,920 min post-tDCS and included data from 16 studies (n = 277 participants). There was a significant main effect for stimulation intensity following tDCS (F_29,539_ = 19.69, *p* < 0.001, Fig. 4, Appendix M, Appendix N). There was an increase in the MEP-to-baseline ratio with intensities ≥ 1.5 mA. The MEP-to-baseline ratio was positively associated with age (*p* < 0.001) and stimulation duration (*p* < 0.001), but not health status (*p* = 0.690). Pooled study estimates are presented in Appendix N, demonstrating heterogeneity (Q = 1881.73, *p* < 0.001, I^2^ = 97.4). The certainty of the evidence was ‘very-low’ after being downgraded for risk of bias, small sample bias and inconsistency.

#### Cathodal tDCS

The effect of stimulation intensity for cathodal tDCS on MEP-to-baseline ratio was evaluated from immediately post-tDCS to 1,920 min post-tDCS and included data from 8 studies (n = 143 participants). There was a significant main effect for stimulation intensity (F_26,189_ = 9.94, *p* < 0.001, Fig. 4, Appendix N, Appendix O). There was no clear directional change based on stimulation intensity. MEP-to-baseline ratio was not associated with age (*p* = 0.980) or stimulation duration (*p* = 0.120). Pooled study estimates are presented in Appendix N, demonstrating heterogeneity (Q = 663.58, *p* < 0.001, I^2^ = 74.6). The certainty of the evidence was ‘very-low’ after being downgraded for risk of bias, small sample bias and inconsistency.

### Stimulation frequency

#### Anodal tDCS

**Fig. 5:**
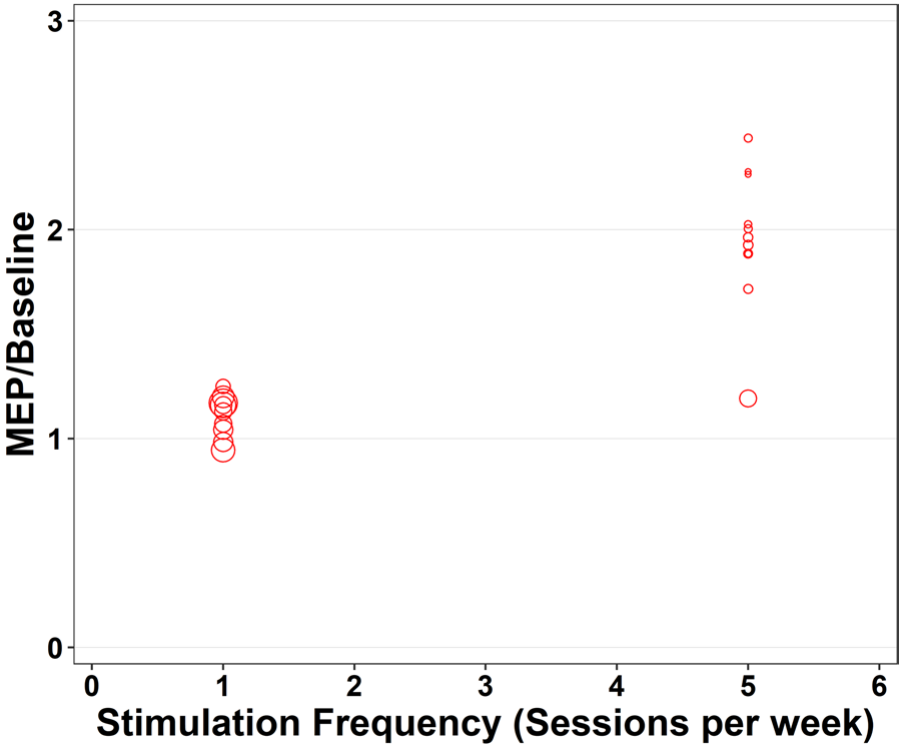
Individual study level effects of anodal transcranial direct current stimulation over time (Stimulation Frequency)

The effect of stimulation frequency for anodal tDCS on MEP-to-baseline ratio was evaluated from immediately post-tDCS to 120 min post-tDCS and included data from 2 studies (n = 23 participants). There was a significant main effect for stimulation frequency (F_10,10_ = 6.379, *p* = 0.004, Fig. 5, Appendix P, Appendix Q). Greater MEP-to-baseline ratios were observed with higher weekly application frequencies. Pooled study estimates are presented in Appendix Q, demonstrating moderate heterogeneity in the I^2^ (Q = 12.94, *p* = 0.227, I^2^ = 78.3). The certainty of the evidence was ‘very-low’ after being downgraded for risk of bias, small sample bias and inconsistency.

#### Cathodal tDCS

The effect of stimulation frequency for cathodal tDCS on MEP-to-baseline ratio was not evaluated as no data were identified.

### Electrode montage

#### Anodal tDCS

**Fig. 6:**
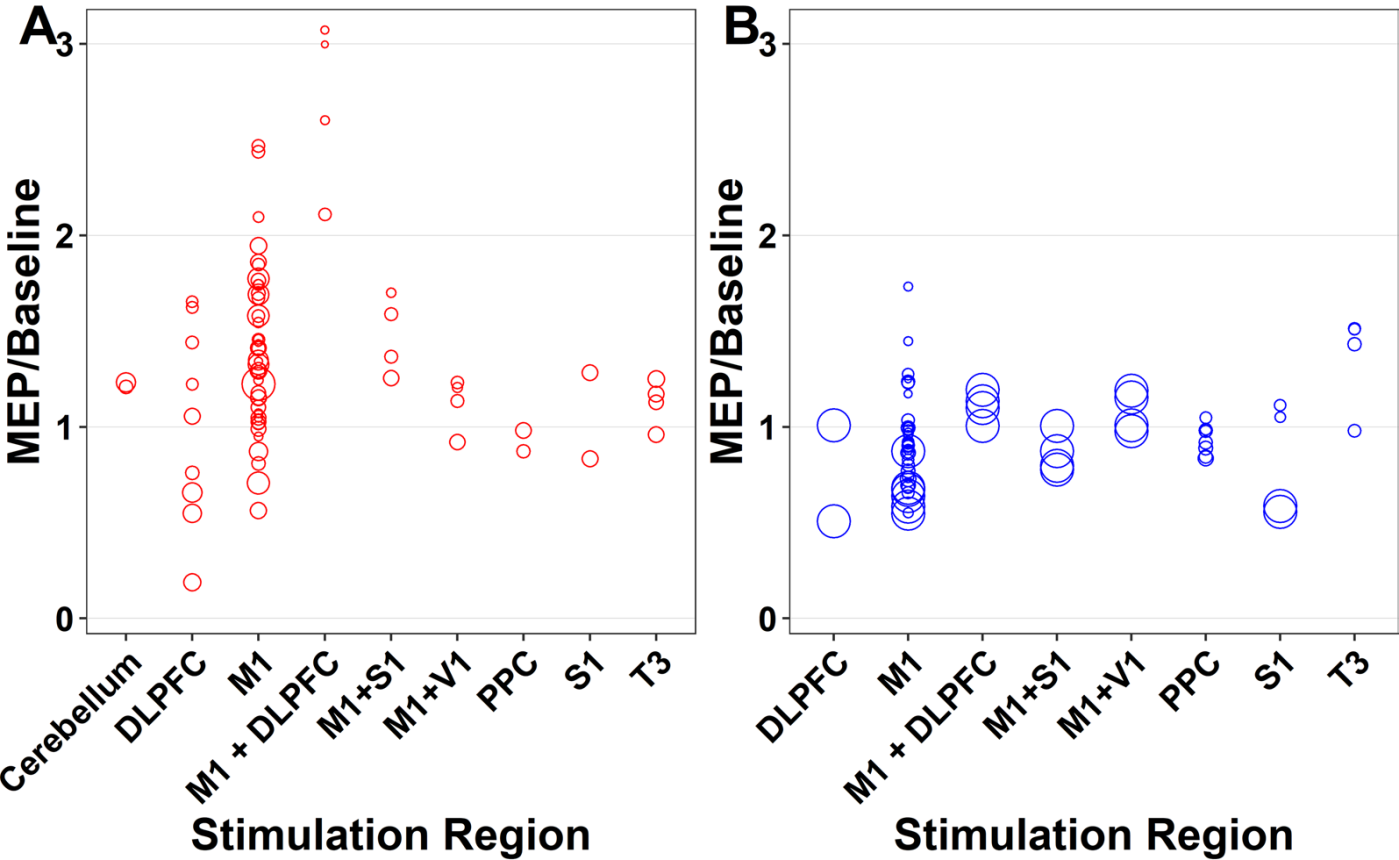
Individual study level effects of anodal transcranial direct current stimulation over time (Electrode Montage): (**A**) Anodal tDCS, (**B**) Cathodal tDCS

The effect of electrode montage for anodal tDCS on MEP-to-baseline ratio was evaluated from immediately post-tDCS to 1,440 min post-tDCS and included data from 16 studies (n = 240 participants). There was a significant main effect for montage locations (F_19,57_ = 17.84, *p* < 0.001, Fig. 6, Appendix R, Appendix S). Cerebellar, primary motor cortex (M1) and dorsolateral pre-frontal cortex (DLPFC) montages performed better than primary somatosensory cortex (S1) or Temporal (T) montages. MEP-to-baseline ratio was positively associated with age (*p* < 0.001), negatively associated with stimulation intensity (*p* = 0.08), but not associated with health status (*p* = 0.14). Pooled study estimates are presented in Appendix S, demonstrating heterogeneity (Q = 700.74, *p* < 0.001, I^2^ = 93.8). The certainty of the evidence was ‘very-low’ after being downgraded for risk of bias, small sample bias and inconsistency.

#### Cathodal tDCS

The effect of electrode montage for cathodal tDCS on MEP-to-baseline ratio was evaluated from immediately post-tDCS to 1,440 min post-tDCS and included data from 9 studies (n = 154 participants). There was a significant main effect for montage locations regions (F_19,45_ = 17.84, *p* < 0.001, Fig. 6, Appendix S, Appendix T). There were no estimated differences observed for the MEP-to-baseline ratio based on montage location. The MEP-to-baseline ratio was positively associated with age (*p* < 0.001), negatively associated with stimulation intensity (*p* = 0.08), and not associated with health status (*p* = 0.14). Pooled study estimates are presented in Appendix S, demonstrating heterogeneity (Q = 802.54, *p* < 0.001, I^2^ = 99.2). The certainty of the evidence was ‘very-low’ after being downgraded for risk of bias, small sample bias and inconsistency.

### Electrode design (e.g., size or shape)

#### Anodal tDCS

The effect of electrode design for anodal tDCS on MEP-to-baseline ratio were evaluated from immediately post-tDCS to 360 min post-tDCS and included data from 2 studies (n = 29 participants). There was a significant main effect for electrode design (F_12,12_ = 27.59, *p* < 0.001, Appendix U and Appendix V). There were significant effects for 4 cm^2^ ring and rectangular 35 cm^2^ montage locations but not rectangular 25 cm^2^ electrodes. Individual study estimates are presented in Appendix W, demonstrating heterogeneity (Q = 161.44, *p* < 0.001, I^2^ = 0). The certainty of the evidence was ‘very-low’ after being downgraded for risk of bias, small sample bias and publication bias.

#### Cathodal tDCS

The effect of electrode design for cathodal tDCS on MEP-to-baseline ratio were evaluated from immediately post-tDCS to 360 min post-tDCS and included data from 2 studies (n = 29 participants). There was a significant main effect for electrode design (F_12,12_ = 38.75, *p* < 0.001, Appendix W, Appendix X). The effect for all montage locations crossed 1 (i.e., no mean change), however there was a trend towards the rectangular 25 cm^2^ electrode montage providing the greatest effect. Individual study estimates are presented in Appendix W, demonstrating heterogeneity (Q = 99.34, *p* < 0.001, I^2^ = 98.8). The certainty of the evidence was ‘very-low’ after being downgraded for risk of bias, small sample bias and inconsistency.

## Discussion

This study used multi-level longitudinal meta-analyses to examine the temporal effects of tDCS parameters on corticospinal excitability; understanding the influence of specific stimulation parameters on tDCS-induced changes in corticospinal excitability could aid researchers and clinicians in making evidence-based decisions regarding tDCS parameters selection. Specifically, this review provides guidance on how to select the polarity, intensity, duration, frequency, montage, and electrode shape/size of tDCS devices in a clinical setting. Whilst the number of participants for each meta-analysis varies, this review includes data from 84 distinct cohorts inclusive of 1,709 participants. This represents a significant advancement over typical studies in the neuroscience field, which often rely on smaller sample sizes (e.g., the median sample size in this review was 15 participants), limiting the generalisability of their findings. [113]

This review supports prior studies that anodal tDCS increases MEP size, whereas cathodal tDCS reduces MEP size [114]. However, unlike previous tDCS reviews [114, 115], we modelled data longitudinally providing more robust estimates on the temporal effects of tDCS over time as we could specifically compare outcomes at each timepoint as opposed to selecting only single (often averaged) time-point comparisons. Our results demonstrated that anodal tDCS appears to increase MEP size for at least 120 min, whereas cathodal tDCS appears to have a shorter duration of effect, reducing MEP size for at least 15 min. Further, our results indicate that stimulation duration appears to be more strongly associated with MEP size change than stimulation intensity. This has clear implications for both clinical practice and research using tDCS (e.g., timing must be considered for anodal versus cathodal tDCS when using tDCS as an adjunct to skill acquisition).

The reported stimulation duration and stimulation intensity for tDCS has varied within studies and there has been little consistency in what parameters should be used [116]. For example, one study (n = 14) reported that anodal tDCS durations beyond 30 min have no effect on cortical excitability [107], whereas the pooled estimates from this review suggest similar effect sizes beyond 20 min. Even implementation guides for parameter selection highlight the variety of methods used without being able to provide specific recommendations [117]. However, this review demonstrated that the required stimulation duration and intensity for anodal and cathodal tDCS were not the same: for anodal tDCS, a stimulation duration of ≥ 20 min appears to be needed to increase MEP size. There were large effects demonstrated with 22 and 24 min, however there was limited data for these timepoints and it seems unlikely to be a true physiological effect when balanced against the other stimulation durations > 20 min. In contrast, cathodal tDCS requires at least 9 min to achieve a reduction in MEP size, with much narrower confidence intervals than anodal-tDCS, thus providing greater confidence in the findings. Anodal tDCS intensities of > 1.5 mA produced consistently larger increases in excitability than lower intensities. However, even low-intensity anodal tDCS (≥ 0.2 mA) demonstrated an effect, suggesting evidence that the observed changes in excitability are unlikely to be solely related to intensity. Alternatively, cathodal tDCS showed no clear intensity-response characteristics. This may be, in part, due to distinct mechanisms of long-term potentiation like plasticity induced via anodal tDCS and long-term depression-like plasticity induced via cathodal tDCS. Regardless, in the absence of clear duration or intensity-response characteristics, we would suggest the minimum duration and intensity parameters that we demonstrated induce MEP size change represent the optimal tDCS parameters as they optimise safety (given they are minimum, yet effective parameters [118]).

While few studies have explored the effects of tDCS frequency, our meta-analysis supports the notion that more frequent applications per week lead to greater changes in MEP size. This aligns with the findings by Alonzo et al. (2012) that demonstrated significant increases in excitability when anodal tDCS was applied for 5 consecutive days. This is clinically important since repeated application of tDCS are more common in clinical settings [119–121]; yet nearly all studies have only implemented a single tDCS intervention, limiting their practical and clinical applicability. Another consideration in the variability of responses, not included in this review is the homogeneity of the electric field generated by tDCS. A number of pathoanatomical variables (e.g., thickness of the cerebrospinal fluid), are known to influence the electrical field generated by tDCS on an individual level [122]. It has also been demonstrated that more focal stimulation results in larger intra-individual variability [25], with the benefit of personalising the electrical field for individuals not currently supported to achieve an effect on MEP size [123].

With clearer direction regarding parameters, future research should also consider user comfort in tDCS devices design and stimulation parameters. Qualitative research has demonstrated that existing devices are difficult to use and unlikely to interest the patient, rehabilitation provider, or athletes outside of controlled laboratory environments without further consideration to design and application that is centred around the end-user [124].

### Limitations

The primary limitation of this longitudinal meta-analysis is the certainty of the evidence. All meta-analyses were assigned ‘very-low’ certainty evidence, typically due to risk of bias (96% of included studies being low quality), small sample bias (median sample size in this review was 15 participants) and inconsistency (substantial heterogeneity within included studies). Whilst, longitudinal meta-analysis does generate an overall effect size (Appendix H), the confidence intervals for the effect sizes at each included timepoint are typically much broader due to smaller samples. This approach is considered superior to standard meta-analysis as the overall estimate of the effect adjusts for each timepoint as opposed to averaging them. Further, when interpreting results by time it is important to not just look at the p-value as some timepoints are likely underpowered and instead look at the effect size and confidence intervals to understand the temporal nature of the intervention.

## Conclusion

The current meta-analyses have provided clear guidance for the selection of parameters when using tDCS. When applied with appropriate parameters, anodal tDCS is generally associated with increased excitability, whereas cathodal tDCS is associated with decreased excitability. Anodal-tDCS appears to increase MEP size for at least 120 min, whereas cathodal tDCS reduces MEP size for at least 15 min. Anodal stimulation duration of ≥ 20 min produces the most consistent effects on MEP size. Stimulation intensities of > 1.5 mA were consistently better than lower intensities at changing MEP size. More than 1 session of tDCS per week creates greater potential for changes in MEP. Different electrode montages are likely to alter MEP size. Finally, larger electrodes appear to be superior to smaller electrodes. We would suggest the minimum, yet effective, parameters for tDCS from this review be adopted to ensure effectiveness and optimise safety.

## Supplementary Information

Additional file1 (DOCX 2529 KB)

Additional file2 (XLSX 49 KB)

## Data Availability

All data generated or analysed during this study are included in this published article [and its supplementary information files].
