# Supplementary material for: What are the optimal transcranial direct current stimulation parameters and design elements to modulate corticospinal excitability? A systematic review and longitudinal meta-analysis
Source: Neurol Res Pract. 2025 Nov 11;7(1):86. doi: 10.1186/s42466-025-00449-1 (PMC12606828; doi:10.1186/s42466-025-00449-1)

**Appendix A. Search strategy**

| **Number** | **Combiners** | **Region** | **Terms** |
| --- | --- | --- | --- |
| 1 | Problem of Interest | Title/ abstract | transcranial direct current stimulation OR tDCS OR transcranial electrical stimulation |
| 2 | Outcome | All | Transcranial magnetic stimulation OR motor evoked potential OR motor threshold OR intracortical inhibition OR silent period OR intracortical facilitation OR corticomedullary evoked potentials OR magnetic resonance imaging OR TMS evoked potential |
| 3 |  |  | #1 AND #2 |
|  | Limitations |  | Human |

**Appendix B. Quality assessment criteria**

| **Bias** | **Item Number** | **Item Definition** | **Criteria for a judgement of ‘YES’** |
| --- | --- | --- | --- |
| Bias related to selection and allocation | 1 | Was true randomization used for assignment of participants to treatment groups? | Method for randomisation clearly described and utilised. |
|  | 2 | Was allocation to treatment groups concealed? | Method for allocation concealment clearly described and utilised. |
|  | 3 | Were treatment groups similar at the baseline? | No between group differences in demographic or outcomes present at baseline. |
| Bias related to administration of intervention/ exposure. | 4 | Were participants blind to treatment assignment? | Method for participant blinding clearly described and utilised. |
|  | 5 | Were those delivering the treatment blind to treatment assignment? | Method for blinding tDCS intervention clearly described and utilised. |
|  | 6 | Were treatment groups treated identically other than the intervention of interest? | No differences in treatment between groups, other than the intervention. |
| Bias related to assessment, detection and measurement of the outcome | 7 | Were outcome assessors blind to treatment assignment? | Method for outcome assessor blinding clearly described and utilised. |
|  | 8 | Were outcomes measured in the same way for treatment groups? | No difference in transcranial magnetic stimulation assessment between groups. |
|  | 9 | Were outcomes measured in a reliable way? | Methods for transcranial magnetic stimulation assessment were clearly described and in accordance with international recommendations for assessment (e.g., data were normalised). |
| Bias related to participant retention | 10 | Was follow up complete and if not, were differences between groups in terms of their follow up adequately described and analysed? | Substantial differences do not exist (>20% difference) in the drop-out rate between groups. |
| Statistical Conclusion Validity | 11 | Were participants analysed in the groups to which they were randomized? | Participants were analysed in the groups to which they were randomized. |
|  | 12 | Was appropriate statistical analysis used? | A statistical model to assess between group differences, with outcomes normalised, was performed. |
|  | 13 | Was the trial design appropriate and any deviations from the standard RCT design (individual randomization, parallel groups) accounted for in the conduct and analysis of the trial? | Reviewer judgement. |

**Appendix C. Extracted TMS Variables other than MEP-to-baseline ratio**

**Appendix D. Individual meta-analysis model methodology**

The specific methodology for each meta-analysis are as follows:

1. Stimulation polarity (Anodal vs Cathodal): this analysis aimed to examine the effects of anodal and cathodal on MEP-to-baseline ratio. The model included stimulation polarity (categorical), population (healthy and clinical), participant mean age (continuous), stimulation intensity, stimulation duration, and time of measurements post-tDCS (ordinal) as moderators. Time contrasts were appropriately adjusted to preserve the ordinal nature of the data. Random intercepts were included at the study level and nested within polarity to account for the hierarchical clustering (~1|study_id/polarity).

2. Stimulation duration: Separate meta-analyses were fitted for anodal and cathodal tDCS to examine the effect of stimulation duration on MEP-to-baseline ratio. For anodal tDCS, the model included stimulation duration, time, population, age, and stimulation intensity. The cathodal tDCS model included the same moderators, except for population as it was excluded due to limited data on clinical population (i.e., studies were only conducted in healthy people). In both analyses, stimulation duration and time were treated as ordinal factors, and contrasts were appropriately adjusted to preserve the ordinal nature of the data. Random intercepts were included to account for repeated measures (~1|study_id).

3. Stimulation intensity (intensity): Separate meta-analyses were conducted for anodal and cathodal tDCS to examine the effect of stimulation intensity on MEP-to-baseline ratio. In the anodal tDCS model, moderators included stimulation intensity, time, stimulation duration, population, and age. The cathodal tDCS model included the same moderators, except population. In both models, stimulation intensity and time were treated as ordinal factors with contrasts adjusted to reflect ordinal structure. Random intercepts were included to account for repeated measures (~1|study_id).

4. Stimulation frequency: This analysis focussed solely on anodal tDCS studies as no relevant cathodal studies were available. We examined the effect of stimulation frequency on MEP-to-baseline ratio, with stimulation frequency and time included as moderators. Both were treated as ordinal factors, and contrasts were adjusted accordingly. Random intercept for study was included in the model to account for repeated measures.

5. Electrode montage (electrode placement): Separate meta-analyses were conducted for anodal and cathodal tDCS to examine the effect of electrode montage on MEP-to-baseline ratio. For both stimulation types, the models included the following moderators: region of stimulation, stimulation current (unilateral or bilateral), stimulation intensity, population, age, and time, and contrasts were adjusted accordingly. Random intercepts for study were included to account for within-study repeated measures.

6. Electrode design (e.g., size and shape): Due to limited number of studies examining the independent effects of electrode size and shape, we combined these factors into a single variable: electrode design. Separate meta-analyses for anodal and cathodal tDCS were then conducted to examine the effect of electrode design on MEP-to-baseline ratio. The models included electrode design and time as moderators, with time treated as an ordinal variable and contrasts adjusted accordingly. Random intercepts for study were included in both models to account for repeated measures.

**Appendix E. Search Strategy Documentation**

| **Source** | **Date of search** | **Search strategy used (keywords & Boolean)** | **Search Limits or filters (e.g. dates, language)** | **# results found** | **Comments** |
| --- | --- | --- | --- | --- | --- |
| PUBMED | 24/11/2023 | ((transcranial direct current stimulation[Title/Abstract] OR tDCS[Title/Abstract] OR transcranial electrical stimulation[Title/Abstract]) AND (Transcranial magnetic stimulation OR motor evoked potential OR motor threshold OR intracortical inhibition OR silent period OR intracortical facilitation OR corticomedullary evoked potentials OR magnetic resonance imaging OR TMS evoked potential)) NOT (Review[Filter]) | Humans | 1765 | Exported to End Note |
| CINAHL (Full Text) | 24/11/2023 | AB ( transcranial direct current stimulation OR tDCS OR transcranial electrical stimulation ) AND TX ( Transcranial magnetic stimulation OR motor evoked potential OR motor threshold OR intracortical inhibition OR silent period OR intracortical facilitation OR corticomedullary evoked potentials OR magnetic resonance imaging OR TMS evoked potential ) NOT PT Review | Humans | 342 | Exported to End Note |
| EBSCO (Medline) | 24/11/2023 | AB ( transcranial direct current stimulation OR tDCS OR transcranial electrical stimulation ) AND TX ( Transcranial magnetic stimulation OR motor evoked potential OR motor threshold OR intracortical inhibition OR silent period OR intracortical facilitation OR corticomedullary evoked potentials OR magnetic resonance imaging OR TMS evoked potential ) NOT PT Review | Humans | 1957 | Exported to End Note |
| Cochrane Library (including clinicaltrials.gov and International Clinical Trials Registry Platform) | 24/11/2023 | transcranial direct current stimulation OR tDCS OR transcranial electrical stimulation in Title Abstract Keyword AND Transcranial magnetic stimulation OR motor evoked potential OR motor threshold OR intracortical inhibition OR silent period OR intracortical facilitation OR corticomedullary evoked potentials OR magnetic resonance imaging OR TMS evoked potential in All Text NOT Review in Publication Type |  | 2120 | Exported to End Note |
| SportsDISCUS | 24/11/2023 | AB ( transcranial direct current stimulation OR tDCS OR transcranial electrical stimulation ) AND TX ( Transcranial magnetic stimulation OR motor evoked potential OR motor threshold OR intracortical inhibition OR silent period OR intracortical facilitation OR corticomedullary evoked potentials OR magnetic resonance imaging OR TMS evoked potential ) NOT TI Review |  | 142 | Exported to End Note |
| Web of Science | 24/11/2023 | ((TI=(transcranial direct current stimulation OR tDCS OR transcranial electrical stimulation)) AND ALL=(Transcranial magnetic stimulation OR motor evoked potential OR motor threshold OR intracortical inhibition OR silent period OR intracortical facilitation OR corticomedullary evoked potentials OR magnetic resonance imaging OR TMS evoked potential)) NOT DT=(Review) |  | 1542 | Exported to End Note |
| Key Journals |  |  |  |  | N/A |
| TOTAL | | | | | 7868 |

**Appendix F. Full-Text Exclusion List**

| **Year** | **Title** | **Exclusion Reason** |
| --- | --- | --- |
| 2009 | Impact of transcranial direct current stimulation on spinal network excitability in humans | Conference abstract |
| 2012 | The effect of transcranial direct current stimulation on myofascial trigger pain syndrome | Conference abstract |
| 2012 | Potential mechanisms underlying the neural effects of TDCS in post-stroke recovery | Conference abstract |
| 2014 | Intensity-dependent effects of tDCS on corticospinal excitability in chronic SCI | Conference abstract |
| 2014 | Neuromodulation of parietal and motor activity affects motor planning and execution | Conference abstract |
| 2015 | Enhancement of motor learning with transcranial direct current stimulation in healthy children | Conference abstract |
| 2015 | Optimized multielectrode tDCS modulates corticolimbic networks: a placebo-controlled crossover study | Conference abstract |
| 2015 | Suppression of LTP-like associative plasticity in the human SMA-M1 network by simultaneous tDCS | Conference abstract |
| 2015 | Transcranial direct current stimulation improves tactile discrimination in stroke patients | Conference abstract |
| 2015 | High-definition tDCS modulates activation during spatial navigation in healthy older adults | Conference abstract |
| 2015 | Cathodal-tDCS induced reduction in excitability of superficial pain neuromatrix cortices is associated with sensory and pain threshold increases | Conference abstract |
| 2015 | The effects of different intensities of anodal tDCS on spinal plasticity induced by patterned electrical stimulation | Conference abstract |
| 2016 | Polarity independent suppression of long-term associative plasticity in the human SMA-M1 network by simultaneous tDCS | Conference abstract |
| 2016 | Dorsolateral prefrontal cortex modulates motor resonance during observation of implied motion in a work of art | Conference abstract |
| 2017 | A phase i current escalation study for transcranial direct current stimulation in ischemic stroke patients | Conference abstract |
| 2017 | The effect of high definition tDCS on cognitive control: a behavioral and fMRI study | Conference abstract |
| 2017 | Effects of transcranial direct current stimulation applied to the prefrontal cortex on TMS evoked potentials | Conference abstract |
| 2017 | Evaluation of a novel MRI technique for mapping in-vivo currents and hemodynamic changes during tDCS | Conference abstract |
| 2017 | Non-invasive brain modulation of aberrant networks in Alzheimer's disease | Conference abstract |
| 2017 | Evaluating the effects of model-based optimal bipolar tDCS configurations on cortical excitability | Conference abstract |
| 2017 | Effect of transcranial direct current stimulation on neurocognition, social cognition, and eeg in schizophrenia | Conference abstract |
| 2017 | Optimized multielectrode tDCS modulates corticolimbic networks | Conference abstract |
| 2018 | Effects of different transcranial direct current stimulation devices on motor cortical excitability | Conference abstract |
| 2018 | Prefrontal bipolar versus multichannel tDCS: impact on working memory performance | Conference abstract |
| 2019 | Transcranial direct current stimulation applied to the left dorsolateral prefrontal cortex in smokers modifies cognitive circuits implicated in the nicotine withdrawal syndrome | Conference abstract |
| 2019 | Shifting the balance between hemispheres or single hemisphere stimulation for response inhibition | Conference abstract |
| 2019 | Brain network modulation in alzheimer's disease and behavioral variant frontotemporal dementia with electrical stimulation: a pilot double-blind randomized trial | Conference abstract |
| 2019 | Impact of Corticospinal Integrity on the Effects of tDCS | Conference abstract |
| 2020 | Multifocal tDCS modulates resting-state functional connectivity in the elderly depending on baseline connectivity and induced electric field | Conference abstract |
| 2020 | Effect of scalp cooling on cortical excitability promoted by transcranial direct current stimulation in healthy subjects | Conference abstract |
| 2020 | Measuring the Effect of Transcranial Direct Current Stimulation (tDCS) on Large-Scale Brain Networks With Simultaneous Functional Magnetic Resonance Imaging (fMRI) | Conference abstract |
| 2020 | Effects of electrode orientation on the impact of transcranial direct current stimulation on motor cortex excitability | Conference abstract |
| 2020 | Transcranial alternating current stimulation does not change the common input to the motor neuron pool | Conference abstract |
| 2020 | Massed vs. distributed tDCS application during motor skill acquisition | Conference abstract |
| 2020 | Mind vestibular off-target effects during Transcranial Direct Current Stimulation! | Conference abstract |
| 2020 | Investigating the therapeutic value of transcranial Direct Current Stimulation on language disorders in the semantic variant of Primary Progressive Aphasia | Conference abstract |
| 2021 | Structural Changes Induced by Transcranial Direct Current Stimulation (tDCS) in Depression | Conference abstract |
| 2022 | PC015 / #972 ONGOING CLINICAL TRIAL ASSESSING THE THERAPEUTIC POTENTIAL OF HIGH-DENSITY PREFRONTAL AND CEREBELLAR TRANSCRANIAL DIRECT CURRENT STIMULATION TO IMPROVE GAIT IN STROKE PATIENTS: E-POSTER VIEWING | Conference abstract |
| 2022 | Transcranial Electrical Stimulation in Meth Craving: a Mechanistic Dose-Response RCT | Conference abstract |
| 2022 | THE EFFECTS OF MONOPOLAR TDCS ON BRAINSTEM REFLEX PATHWAYS | Conference abstract |
| 2022 | P342. Modulation of Depression-Relevant Circuitry by Transcranial Direct Current Stimulation (tDCS) | Conference abstract |
| 2022 | TH-201. Transferability of cathodal tDCS effects from the primary motor to the dorsolateral prefrontal cortex: a multimodal TMS-EEG study | Conference abstract |
| 2023 | Quantitative measurement of changes in 23Na MRI associated with transcranial direct current stimulation (tDCS) of the motor cortex | Conference abstract |
| 2023 | FV 10 Cerebellar modulation of associative plasticity is impaired in cervical dystonia | Conference abstract |
| 2023 | Modelling novel tDCS settings for interfering with the brainstem activity | Conference abstract |
| 2022 | WpÅ‚yw przezczaszkowej stymulacji prÄ…dowej na odczuwanie bÃ³lu i zuÅ¼ycie opioidÃ³w po zabiegach chirurgicznych krÄ™gosÅ‚upa | Full text not available |
| 2009 | Brain polarization enhances the formation and retention of motor memories | No comparator |
| 2013 | Validation of finite element model of transcranial electrical stimulation using scalp potentials: implications for clinical dose | No comparator |
| 2015 | What is the optimal anodal electrode position for inducing corticomotor excitability changes in transcranial direct current stimulation? | No comparator |
| 2016 | Augmenting Cognitive Training In Older Adults | No comparator |
| 2016 | Spatial and polarity precision of concentric high-definition transcranial direct current stimulation (HD-tDCS) | No comparator |
| 2017 | Anodal transcranial direct current stimulation shows minimal, measure-specific effects on dynamic postural control in young and older adults: a double blind, sham-controlled study | No comparator |
| 2017 | Propriospinal cutaneous-induced EMG suppression is unaltered by anodal tDCS of healthy motor cortex | No comparator |
| 2018 | A brain imaging study of the effects of electrical brain stimulation on attention in healthy people | No comparator |
| 2018 | Test-Retest Reliability of Homeostatic Plasticity in the Human Primary Motor Cortex | No comparator |
| 2019 | Effects of posture on electric fields of non-invasive brain stimulation | No comparator |
| 2020 | Preconditioning cathodal transcranial direct current stimulation facilitates the neuroplastic effect of subsequent anodal transcranial direct current stimulation applied during cycling in young adults | No comparator |
| 2016 | Transcranial direct current stimulation improves isometric time to exhaustion of the knee extensors | No comparator |
| 2017 | The effect of transcranial direct current stimulation on motor sequence learning and upper limb function after stroke | No comparator |
| 2021 | Effect of conventional transcranial direct current stimulation devices and electrode sizes on motor cortical excitability of the quadriceps muscle | No comparator |
| 2013 | Formation of cortical plasticity in older adults following tDCS and motor training | No comparator |
| 2010 | Modulation of mu rhythm desynchronization during motor imagery by transcranial direct current stimulation | No comparator |
| 2014 | A dissociation between propriospinal facilitation and inhibition after bilateral transcranial direct current stimulation | No comparator |
| 2021 | Individualization of tDCS intensity according to corticospinal excitability does not improve stimulation efficacy over the primary motor cortex | No comparator |
| 2013 | A Comparison between Uni- and Bilateral tDCS Effects on Functional Connectivity of the Human Motor Cortex | No comparator |
| 2013 | Anodal transcranial direct current stimulation modulates GABAB-related intracortical inhibition in the M1 of healthy individuals | No comparator |
| 2021 | Multifocal stimulation of the cerebro-cerebellar loop during the acquisition of a novel motor skill | No comparator |
| 2023 | High-definition transcranial direct current stimulation for upper extremity rehabilitation in moderate-to-severe ischemic stroke: a pilot study | No comparator |
| 2023 | The interaction between metaplastic neuromodulation and fatigue in multiple sclerosis | No comparator |
| 2017 | Transcranial Direct-Current Stimulation Can Enhance Motor Learning in Children | Paediatric population |
| 2022 | Efficacy of adjunctive intensive transcranial direct current stimulation of different cortices in treatment-resistant depression: a study protocol for a randomized double-blinded sham-controlled trial | Study Protocol |
| 2022 | MRI-Based Personalized Transcranial Direct Current Stimulation to Enhance the Upper Limb Function in Patients with Stroke: study Protocol for a Double-Blind Randomized Controlled Trial | Study Protocol |
| 2012 | Non-invasive cerebellar stimulation for focal dystonia | Trial Registration (No results |
| 2004 | Brain Electrical Stimulation to Enhance Recovery After Stroke | Trial Registration (No results) |
| 2010 | Methods to Enhance Transcranial Direct Stimulation (tDCS) | Trial Registration (No results) |
| 2011 | Can transcranial direct current stimulation (tDCS) improve mirror system activity in autism spectrum disorders? | Trial Registration (No results) |
| 2011 | Effect van hersenstimulatie op de benen | Trial Registration (No results) |
| 2012 | Therapeutic Effect of Direct Current Stimulation on Cognitive Function of Mild to Moderate Alzheimer Patients | Trial Registration (No results) |
| 2014 | Effects of Transcranial Direct Current Stimulation Associated With Physical Exercise: a Metaplasticity Study | Trial Registration (No results) |
| 2014 | Analgesic Effect of Cathodal tDCS Over Right DLPFC in Subjects With Muscular TMD: a Double Blind Crossover RCT | Trial Registration (No results) |
| 2014 | tDCS in Cervical Dystonia | Trial Registration (No results) |
| 2017 | Optimizing Transcranial Direct Current Stimulation for Motor Recovery From Hemiparesis | Trial Registration (No results) |
| 2017 | Influence of brain activation by transcranial direct current electrical stimulation on sensation of the hand | Trial Registration (No results) |
| 2018 | Do brain measures predict improvement of hand or arm function following brain stimulation in people with stroke? | Trial Registration (No results) |
| 2018 | Functional evaluation of motor cortex in healthy subjects with paired synchronous electromagnetic stimulation over the same position | Trial Registration (No results) |
| 2018 | Modulation of Spontaneous Cortical Activity by tDCS: BRAIN Initiative I | Trial Registration (No results) |
| 2018 | Most Effective Stimulation Site in Transcranial Direct Current Stimulation for Gait Recovery After Stoke | Trial Registration (No results) |
| 2018 | Establish the optimum tDCS application on gait performance and brain activity -Exploration of people with chronic stroke | Trial Registration (No results) |
| 2019 | Cerebral Blood Flow and tDCS | Trial Registration (No results) |
| 2019 | Noninvasive Brain Stimulation to Enhance Cognitive Training in Older Adults | Trial Registration (No results) |
| 2019 | Improving Insulin Sensitivity by Non-invasive Brain Stimulation in Persons With Insulin Resistance | Trial Registration (No results) |
| 2020 | Neurophysiological and behavioural effects of a single tDCS treatment in subacute stroke patients | Trial Registration (No results) |
| 2020 | Effect of electric and magnetic brain stimulation on physical performance | Trial Registration (No results) |
| 2020 | Transcranial Direct Current Stimulation Effect on Pain Threshold and Working Memory: impact of Age and Protocol Type | Trial Registration (No results) |
| 2020 | Conventional or High Definition tDCS to Enhance Implicit Motor Sequence Learning in Healthy Young Adults? | Trial Registration (No results) |
| 2021 | The Effect of Unihemispheric Concurrent Dual-Site Transcranial Direct Current Stimulation of Primary Motor and Dorsolateral Prefrontal Cortices on cerebral plasticity (with magnetic resonance spectroscopy) in Patients With chronic Stroke: a double-blind controlled clinical trial | Trial Registration (No results) |
| 2021 | Modulating Prospective Memory in Older Adults With Non-invasive Brain Stimulation | Trial Registration (No results) |
| 2021 | Effect of Transcranial Direct Current Stimulation (TDCS) on the Resistance to Tiredness of the Biceps Brachial Muscle in the Elderly | Trial Registration (No results) |
| 2022 | Enhancing Brain Health by tDCS in Persons With Overweight and Obesity | Trial Registration (No results) |
| 2023 | Investigation The Effect of Conventional Vs. Individualized tDCS Intensity to Achieve Uniform E-Fields | Trial Registration (No results) |
| 2023 | HD-tDCS to Modulate Connectivity | Trial Registration (No results) |
| 2023 | Cortical Excitability Modulation With ctDCS in Fibromyalgia | Trial Registration (No results) |
| 2012 | No effect of a single session of transcranial direct current stimulation on experimentally induced pain in patients with chronic low back pain--an exploratory study | Wrong intervention (Concurrent interventions) |
| 2015 | Inhibitory transcranial direct current stimulation enhances weak beta event-related synchronization after foot motor imagery in patients with lower limb amputation | Wrong intervention (Concurrent interventions) |
| 2018 | The Response of the Primary Motor Cortex to Neuromodulation is Altered in Chronic Low Back Pain: A Preliminary Study | Wrong intervention (Concurrent interventions) |
| 2019 | Intramuscular electrical stimulus potentiates motor cortex modulation effects on pain and descending inhibitory systems in knee osteoarthritis: a randomized, factorial, sham-controlled study | Wrong intervention (Concurrent interventions) |
| 2017 | Acute and chronic effects of noradrenergic enhancement on transcranial direct current stimulation-induced neuroplasticity in humans | Wrong intervention (Pharmacology) |
| 2021 | Examining state-dependent effects of transcranial direct current stimulation on visual search and executive function tasks | Wrong intervention (Pharmacology) |
| 2020 | Gamma-transcranial alternating current stimulation and theta-burst stimulation: inter-subject variability and the role of BDNF | Wrong Intervention (tACS) |
| 2012 | Direct-current-dependent shift of theta-burst-induced plasticity in the human motor cortex | Wrong intervention (TMS) |
| 2017 | 5 kHz transcranial alternating current stimulation: lack of cortical excitability changes when grouped in a theta burst pattern | Wrong intervention (TMS) |
| 2014 | Anodal transcranial pulsed current stimulation: A novel technique to enhance corticospinal excitability | Wrong Intervention (tPCS) |
| 2015 | Anodal Transcranial Pulsed Current Stimulation: the Effects of Pulse Duration on Corticospinal Excitability | Wrong Intervention (tPCS) |
| 2014 | Effects of transvertebral direct current stimulation in healthy humans: early results from an ongoing randomized cross over study | Wrong Intervention (Trans-Spinal) |
| 2014 | Does trans-spinal direct current stimulation alter phrenic motoneurons and respiratory neuromechanical outputs in humans? A double-blind, sham-controlled, randomized, crossover study | Wrong Intervention (Trans-Spinal) |
| 2017 | Higher success rate with transcranial electrical stimulation of motor-evoked potentials using constant-voltage stimulation compared with constant-current stimulation in patients undergoing spinal surgery | Wrong Intervention (Trans-Spinal) |
| 2018 | Dose-response relationship of transcutaneous spinal direct current stimulation in healthy humans: A proof-of-concept study | Wrong Intervention (Trans-Spinal) |
| 2018 | Neuromodulation of lower limb motor responses with transcutaneous lumbar spinal cord direct current stimulation | Wrong Intervention (Trans-Spinal) |
| 2019 | Cervical trans-spinal direct current stimulation: a modelling-experimental approach | Wrong Intervention (Trans-Spinal) |
| 2022 | Blinding efficacy and adverse events following repeated transcranial alternating current, direct current, and random noise stimulation | Wrong intervention (tRNS) |
| 2006 | Transient tinnitus suppression induced by repetitive transcranial magnetic stimulation and transcranial direct current stimulation | Wrong outcome (Clinical Outcomes) |
| 2006 | Testing for causality with transcranial direct current stimulation: pitch memory and the left supramarginal gyrus | Wrong outcome (Clinical Outcomes) |
| 2007 | Go-no-go task performance improvement after anodal transcranial DC stimulation of the left dorsolateral prefrontal cortex in major depression | Wrong outcome (Clinical Outcomes) |
| 2007 | Diminishing risk-taking behavior by modulating activity in the prefrontal cortex: a direct current stimulation study | Wrong outcome (Clinical Outcomes) |
| 2007 | Activation of prefrontal cortex by transcranial direct current stimulation reduces appetite for risk during ambiguous decision making | Wrong outcome (Clinical Outcomes) |
| 2007 | Site-specific effects of transcranial direct current stimulation on sleep and pain in fibromyalgia: a randomized, sham-controlled study | Wrong outcome (Clinical Outcomes) |
| 2008 | Modulatory effects of anodal transcranial direct current stimulation on perception and pain thresholds in healthy volunteers | Wrong outcome (Clinical Outcomes) |
| 2008 | Enhancing language performance with non-invasive brain stimulation--a transcranial direct current stimulation study in healthy humans | Wrong outcome (Clinical Outcomes) |
| 2008 | Transcranial direct current stimulation improves recognition memory in Alzheimer disease | Wrong outcome (Clinical Outcomes) |
| 2009 | Efficacy of anodal transcranial direct current stimulation (tDCS) for the treatment of fibromyalgia: results of a randomized, sham-controlled longitudinal clinical trial | Wrong outcome (Clinical Outcomes) |
| 2009 | Temporal lobe cortical electrical stimulation during the encoding and retrieval phase reduces false memories | Wrong outcome (Clinical Outcomes) |
| 2009 | Bilateral frontal transcranial direct current stimulation: failure to replicate classic findings in healthy subjects | Wrong outcome (Clinical Outcomes) |
| 2010 | Transcranial direct current stimulation facilitates decision making in a probabilistic guessing task | Wrong outcome (Clinical Outcomes) |
| 2010 | Visual memory improved by non-invasive brain stimulation | Wrong outcome (Clinical Outcomes) |
| 2010 | The truth about lying: inhibition of the anterior prefrontal cortex improves deceptive behavior | Wrong outcome (Clinical Outcomes) |
| 2011 | Transcranial direct current stimulation: electrode montage in stroke | Wrong outcome (Clinical Outcomes) |
| 2011 | Transcranial DC Stimulation in Fibromyalgia: Optimized Cortical Target Supported by High-Resolution Computational Models | Wrong outcome (Clinical Outcomes) |
| 2011 | Cathodal transcranial direct current stimulation of the right Wernicke's area improves comprehension in subacute stroke patients | Wrong outcome (Clinical Outcomes) |
| 2011 | Transcranial direct current stimulation improves word retrieval in healthy and nonfluent aphasic subjects | Wrong outcome (Clinical Outcomes) |
| 2011 | Short- and long-lasting tinnitus relief induced by transcranial direct current stimulation | Wrong outcome (Clinical Outcomes) |
| 2011 | Down-regulation of negative emotional processing by transcranial direct current stimulation: effects of personality characteristics | Wrong outcome (Clinical Outcomes) |
| 2012 | Testing the activation-orientation account of spatial attentional asymmetries using transcranial direct current stimulation | Wrong outcome (Clinical Outcomes) |
| 2012 | Transcranial direct current stimulation in treatment resistant depression: a randomized double-blind, placebo-controlled study | Wrong outcome (Clinical Outcomes) |
| 2012 | Anodal transcranial direct current stimulation reduces psychophysically measured surround suppression in the human visual cortex | Wrong outcome (Clinical Outcomes) |
| 2012 | When less is more: evidence for a facilitative cathodal tDCS effect in attentional abilities | Wrong outcome (Clinical Outcomes) |
| 2012 | Improving emotional prosody detection in the attending ear by cathodal tDCS suppression of the competing channel | Wrong outcome (Clinical Outcomes) |
| 2012 | tDCS of the primary motor cortex improves the detection of semantic dissonance | Wrong outcome (Clinical Outcomes) |
| 2013 | Postoperative analgesic effect of transcranial direct current stimulation in lumbar spine surgery: a randomized control trial | Wrong outcome (Clinical Outcomes) |
| 2013 | Polarity- and valence-dependent effects of prefrontal transcranial direct current stimulation on heart rate variability and salivary cortisol | Wrong outcome (Clinical Outcomes) |
| 2013 | Electrode montage dependent effects of transcranial direct current stimulation on semantic fluency | Wrong outcome (Clinical Outcomes) |
| 2013 | Focal modulation of the primary motor cortex in fibromyalgia using 4Ã—1-ring high-definition transcranial direct current stimulation (HD-tDCS): immediate and delayed analgesic effects of cathodal and anodal stimulation | Wrong outcome (Clinical Outcomes) |
| 2013 | Anodal transcranial direct current stimulation (tDCS) over supplementary motor area (SMA) but not pre-SMA promotes short-term visuomotor learning | Wrong outcome (Clinical Outcomes) |
| 2013 | Modulating lexical and semantic processing by transcranial direct current stimulation | Wrong outcome (Clinical Outcomes) |
| 2013 | Is motor learning mediated by tDCS intensity? | Wrong outcome (Clinical Outcomes) |
| 2013 | Disrupting prefrontal cortex prevents performance gains from sensory-motor training | Wrong outcome (Clinical Outcomes) |
| 2013 | Anodal tDCS over SMA decreases the probability of withholding an anticipated action | Wrong outcome (Clinical Outcomes) |
| 2013 | Transcranial direct current stimulation accelerates allocentric target detection | Wrong outcome (Clinical Outcomes) |
| 2013 | Right but not left angular gyrus modulates the metric component of the mental body representation: a tDCS study | Wrong outcome (Clinical Outcomes) |
| 2013 | Transcranial direct current stimulation changes human endowment effect | Wrong outcome (Clinical Outcomes) |
| 2014 | Transcranial direct current stimulation over right dorsolateral prefrontal cortex enhances error awareness in older age | Wrong outcome (Clinical Outcomes) |
| 2014 | Efficacy of transcranial direct current stimulation in patients with drug-naive restless legs syndrome | Wrong outcome (Clinical Outcomes) |
| 2014 | Combining transcranial direct current stimulation and tailor-made notched music training to decrease tinnitus-related distress--a pilot study | Wrong outcome (Clinical Outcomes) |
| 2014 | "If two witches would watch two watches, which witch would watch which watch?" tDCS over the left frontal region modulates tongue twister repetition in healthy subjects | Wrong outcome (Clinical Outcomes) |
| 2014 | Something to talk about: enhancement of linguistic cohesion through tdCS in chronic non fluent aphasia | Wrong outcome (Clinical Outcomes) |
| 2015 | Reducing the Disruptive Effects of Interruptions With Noninvasive Brain Stimulation | Wrong outcome (Clinical Outcomes) |
| 2015 | Ipsilesional and contralesional regions participate in the improvement of poststroke aphasia: a transcranial direct current stimulation study | Wrong outcome (Clinical Outcomes) |
| 2015 | Transcranial direct current stimulation on primary sensorimotor area has no effect in patients with drug-naive restless legs syndrome: a proof-of-concept clinical trial | Wrong outcome (Clinical Outcomes) |
| 2015 | Enhancing Hebbian learning to control brain oscillatory activity | Wrong outcome (Clinical Outcomes) |
| 2015 | Modulation of neural activity in the temporoparietal junction with transcranial direct current stimulation changes the role of beliefs in moral judgment | Wrong outcome (Clinical Outcomes) |
| 2015 | Cerebellar direct current stimulation enhances on-line motor skill acquisition through an effect on accuracy | Wrong outcome (Clinical Outcomes) |
| 2015 | Effects of Transcranial Direct Current Stimulation (tDCS) on Pain Distress Tolerance: A Preliminary Study | Wrong outcome (Clinical Outcomes) |
| 2016 | The impact of transcranial direct current stimulation (tDCS) combined with modified constraint-induced movement therapy (mCIMT) on upper limb function in chronic stroke: a double-blind randomized controlled trial | Wrong outcome (Clinical Outcomes) |
| 2016 | Stimulation of Dorsolateral Prefrontal Cortex Enhances Adaptive Cognitive Control: a High-Definition Transcranial Direct Current Stimulation Study | Wrong outcome (Clinical Outcomes) |
| 2016 | Safety, Tolerability, Blinding Efficacy and Behavioural Effects of a Novel MRI-Compatible, High-Definition tDCS Set-Up | Wrong outcome (Clinical Outcomes) |
| 2016 | No significant effect of transcranial direct current stimulation (tDCS) found on simple motor reaction time comparing 15 different simulation protocols | Wrong outcome (Clinical Outcomes) |
| 2016 | Enhanced motor learning with bilateral transcranial direct current stimulation: impact of polarity or current flow direction? | Wrong outcome (Clinical Outcomes) |
| 2016 | Repetitive transcranial direct current stimulation induced excitability changes of primary visual cortex and visual learning effects: A pilot study | Wrong outcome (Clinical Outcomes) |
| 2016 | Cerebellar tDCS as a novel treatment for aphasia? Evidence from behavioral and resting-state functional connectivity data in healthy adults | Wrong outcome (Clinical Outcomes) |
| 2016 | tDCS over left M1 or DLPFC does not improve learning of a bimanual coordination task | Wrong outcome (Clinical Outcomes) |
| 2016 | Effects of left primary motor and dorsolateral prefrontal cortex transcranial direct current stimulation on laser-evoked potentials in migraine patients and normal subjects | Wrong outcome (Clinical Outcomes) |
| 2016 | Polarity-Specific Cortical Effects of Transcranial Direct Current Stimulation in Primary Somatosensory Cortex of Healthy Humans | Wrong outcome (Clinical Outcomes) |
| 2016 | Source memory performance is modulated by transcranial direct current stimulation over the left posterior parietal cortex | Wrong outcome (Clinical Outcomes) |
| 2016 | Perceptual decisions regarding object manipulation are selectively impaired in apraxia or when tDCS is applied over the left IPL | Wrong outcome (Clinical Outcomes) |
| 2016 | Anodal-tDCS over the human right occipital cortex enhances the perception and memory of both faces and objects | Wrong outcome (Clinical Outcomes) |
| 2017 | Improved grasp function with transcranial direct current stimulation in chronic spinal cord injury | Wrong outcome (Clinical Outcomes) |
| 2017 | Anodal transcranial direct current stimulation over the right hemisphere improves auditory comprehension in a case of dementia | Wrong outcome (Clinical Outcomes) |
| 2017 | Anodal cerebellar tDCS modulates lower extremity pain perception | Wrong outcome (Clinical Outcomes) |
| 2017 | Transcranial Stimulation Over the Dorsolateral Prefrontal Cortex Increases the Impact of Past Expenses on Decision-Making | Wrong outcome (Clinical Outcomes) |
| 2017 | Cathodal transcranial direct current stimulation (tDCS) applied to the left premotor cortex (PMC) stabilizes a newly learned motor sequence | Wrong outcome (Clinical Outcomes) |
| 2017 | Polarity-independent effects of transcranial direct current stimulation over the bilateral opercular somatosensory region: a magnetoencephalography study | Wrong outcome (Clinical Outcomes) |
| 2017 | Anodal transcranial direct current stimulation of right temporoparietal area inhibits self-recognition | Wrong outcome (Clinical Outcomes) |
| 2017 | Cerebellar tDCS Effects on Conditioned Eyeblinks using Different Electrode Placements and Stimulation Protocols | Wrong outcome (Clinical Outcomes) |
| 2017 | Transcranial direct current stimulation of the medial prefrontal cortex dampens mind-wandering in men | Wrong outcome (Clinical Outcomes) |
| 2017 | Effects of transcranial direct current stimulation on steady-state visual evoked potentials | Wrong outcome (Clinical Outcomes) |
| 2017 | Extending the limits of force endurance: Stimulation of the motor or the frontal cortex? | Wrong outcome (Clinical Outcomes) |
| 2017 | Modulating the assessment of semantic speech-gesture relatedness via transcranial direct current stimulation of the left frontal cortex | Wrong outcome (Clinical Outcomes) |
| 2017 | Differential sensory cortical involvement in auditory and visual sensorimotor temporal recalibration: Evidence from transcranial direct current stimulation (tDCS) | Wrong outcome (Clinical Outcomes) |
| 2018 | Manipulating laterality and polarity of transcranial direct current stimulation to optimise outcomes for anomia therapy in an individual with chronic Brocas aphasia | Wrong outcome (Clinical Outcomes) |
| 2018 | Anodal transcranial direct current stimulation enhances positive changes in movement functions, visual attention and depression of patients with chronic ischemic stroke: a clinical trial | Wrong outcome (Clinical Outcomes) |
| 2018 | Patient-delivered tDCS on chronic neuropathic pain in prior responders to TMS (A randomized controlled pilot study) | Wrong outcome (Clinical Outcomes) |
| 2018 | Effects of single versus dual-site High-Definition transcranial direct current stimulation (HD-tDCS) on cortical reactivity and working memory performance in healthy subjects | Wrong outcome (Clinical Outcomes) |
| 2019 | The role of ventromedial prefrontal cortex and temporo-parietal junction in third-party punishment behavior | Wrong outcome (Clinical Outcomes) |
| 2019 | Dissociable Roles within the Social Brain for Self-Other Processing: a HD-tDCS Study | Wrong outcome (Clinical Outcomes) |
| 2019 | Maximizing the Treatment Benefit of tDCS in Neurodegenerative Anomia | Wrong outcome (Clinical Outcomes) |
| 2019 | Efficiency of Repetitive Transcranial Direct Current Stimulation of the Dorsolateral Prefrontal Cortex in Disorders of Consciousness: a Randomized Sham-Controlled Study | Wrong outcome (Clinical Outcomes) |
| 2019 | Sex-based differences in right dorsolateral prefrontal cortex roles in fairness norm compliance | Wrong outcome (Clinical Outcomes) |
| 2019 | Cognitive enhancement with Salience Network electrical stimulation is influenced by network structural connectivity | Wrong outcome (Clinical Outcomes) |
| 2019 | Monophasic transcranial constant-current versus constant-voltage stimulation of motor-evoked potentials during spinal surgery | Wrong outcome (Clinical Outcomes) |
| 2019 | Does Transcranial Direct Current Stimulation Affect the Learning of a Fine Sequential Hand Motor Skill with Motor Imagery? | Wrong outcome (Clinical Outcomes) |
| 2020 | Acute offline transcranial direct current stimulation does not change pain or anxiety produced by the cold pressor test | Wrong outcome (Clinical Outcomes) |
| 2020 | Transcranial Direct Current Stimulation of the Medial Prefrontal Cortex Has No Specific Effect on Self-referential Processes | Wrong outcome (Clinical Outcomes) |
| 2020 | Cerebellar transcranial direct current stimulation in people with parkinsons disease: a pilot study | Wrong outcome (Clinical Outcomes) |
| 2020 | Transcranial direct current stimulation effects on hand sensibility as measured by an objective quantitative analysis device: a randomized single-blind sham-control crossover clinical trial | Wrong outcome (Clinical Outcomes) |
| 2020 | Dose-controlled tDCS reduces electric field intensity variability at a cortical target site | Wrong outcome (Clinical Outcomes) |
| 2020 | Effects of different transcranial direct current stimulation protocols on visuo-spatial contextual learning formation: evidence of homeostatic regulatory mechanisms | Wrong outcome (Clinical Outcomes) |
| 2020 | Neurocognitive effects of transcranial direct current stimulation (tDCS) in unipolar and bipolar depression: Findings from an international randomized controlled trial | Wrong outcome (Clinical Outcomes) |
| 2021 | Transcranial direct current stimulation of the right dorsolateral prefrontal cortex improves response inhibition | Wrong outcome (Clinical Outcomes) |
| 2021 | The influence of tDCS intensity on decision-making training and transfer outcomes | Wrong outcome (Clinical Outcomes) |
| 2021 | Effect of transcranial direct current stimulation on the psychomotor, cognitive, and motor performances of power athletes | Wrong outcome (Clinical Outcomes) |
| 2021 | Effectiveness of unihemispheric concurrent dual-site stimulation over m1 and dorsolateral prefrontal cortex stimulation on pain processing: a triple blind cross-over control trial | Wrong outcome (Clinical Outcomes) |
| 2021 | Effect of anodal high-definition transcranial direct current stimulation on the pain sensitivity in a healthy population: a double-blind, sham-controlled study | Wrong outcome (Clinical Outcomes) |
| 2021 | Verbal Fluency in Mild Alzheimer's Disease: transcranial Direct Current Stimulation over the Dorsolateral Prefrontal Cortex | Wrong outcome (Clinical Outcomes) |
| 2021 | Transcranial direct current stimulation over the left anterior temporal lobe during memory retrieval differentially affects true and false recognition in the DRM task | Wrong outcome (Clinical Outcomes) |
| 2021 | Enhancing creativity by altering the frontoparietal control network functioning using transcranial direct current stimulation | Wrong outcome (Clinical Outcomes) |
| 2021 | Cerebellar tDCS Alters the Perception of Optic Flow | Wrong outcome (Clinical Outcomes) |
| 2021 | The role of dorsolateral and ventromedial prefrontal cortex in the processing of emotional dimensions | Wrong outcome (Clinical Outcomes) |
| 2021 | How social is the cerebellum? Exploring the effects of cerebellar transcranial direct current stimulation on the prediction of social and physical events | Wrong outcome (Clinical Outcomes) |
| 2021 | Ventromedial Prefrontal Cortex Drives the Prioritization of Self-Associated Stimuli in Working Memory | Wrong outcome (Clinical Outcomes) |
| 2022 | Single Session Anodal Transcranial Direct Current Stimulation on Different Cortical Areas: effects on Pain Modulation in Healthy Subjects | Wrong outcome (Clinical Outcomes) |
| 2022 | The effect of high-definition transcranial direct current stimulation on pain processing in a healthy population: a single-blinded crossover controlled study | Wrong outcome (Clinical Outcomes) |
| 2022 | Effects of High-Definition Transcranial Direct Current Stimulation Over the Primary Motor Cortex on Cold Pain Sensitivity Among Healthy Adults | Wrong outcome (Clinical Outcomes) |
| 2022 | Transcranial direct current stimulation of 3 cortical targets is no more effective than placebo as treatment for fibromyalgia: a double-blind sham-controlled clinical trial | Wrong outcome (Clinical Outcomes) |
| 2022 | Anodal Transcranial Direct Current Stimulation Reduces Secondary Hyperalgesia Induced by low Frequency Electrical Stimulation in Healthy Volunteers | Wrong outcome (Clinical Outcomes) |
| 2022 | Effects of Transcranial Direct Current Stimulation (t-DCS) of the Cerebellum on Pain Perception and Endogenous Pain Modulation: a Randomized, Monocentric, Double-Blind, Sham-Controlled Crossover Study | Wrong outcome (Clinical Outcomes) |
| 2022 | Cerebellar Transcranial Direct Current Stimulation Does Not Alter Somatosensory Temporal Discrimination Threshold | Wrong outcome (Clinical Outcomes) |
| 2022 | Non-invasive Brain Stimulation Can Reduce Unilateral Spatial Neglect after Stroke: ELETRON Trial | Wrong outcome (Clinical Outcomes) |
| 2022 | Comparing amplitudes of transcranial direct current stimulation (tDCS) to the sensorimotor cortex during swallowing | Wrong outcome (Clinical Outcomes) |
| 2022 | Effects of transcranial direct current stimulation on cough reflex and urge-to-cough in healthy young adults | Wrong outcome (Clinical Outcomes) |
| 2022 | Effects of transcranial direct current stimulation in pain and opioid consumption after spine surgery | Wrong outcome (Clinical Outcomes) |
| 2022 | Disentangling cognitive inflexibility in major depressive disorder: A transcranial direct current stimulation study | Wrong outcome (Clinical Outcomes) |
| 2022 | Transcranial direct current stimulation of the premotor cortex aimed to improve hand motor function in chronic stroke patients | Wrong outcome (Clinical Outcomes) |
| 2022 | Transcranial Direct Current Stimulation of the Ventromedial Prefrontal Cortex Modulates Perceptual and Neural Patterns of Fear Generalization | Wrong outcome (Clinical Outcomes) |
| 2022 | Effects of tDCS on reward responsiveness and valuation in Parkinson's patients with impulse control disorders | Wrong outcome (Clinical Outcomes) |
| 2022 | Addressing the inconsistent electric fields of tDCS by using patient-tailored configurations in chronic stroke: Implications for treatment | Wrong outcome (Clinical Outcomes) |
| 2022 | Transcranial direct current stimulation (tDCS) modulates motor execution in a limb reaching task | Wrong outcome (Clinical Outcomes) |
| 2022 | Transcranial Direct Current Stimulation Reduces the Negative Impact of Mental Fatigue on Swimming Performance | Wrong outcome (Clinical Outcomes) |
| 2023 | Effect of prefrontal transcranial direct current stimulation on sexual arousal: a proof of concept study | Wrong outcome (Clinical Outcomes) |
| 2023 | The role of left temporo-parietal and inferior frontal cortex in comprehending syntactically complex sentences: A brain stimulation study | Wrong outcome (Clinical Outcomes) |
| 2023 | Electrical stimulation of the cerebellum facilitates automatic but not controlled word retrieval | Wrong outcome (Clinical Outcomes) |
| 2023 | Transcranial Direct Current Stimulation Electrode Montages May Differentially Impact Variables of Walking Performance in Individuals Poststroke: A Preliminary Study | Wrong outcome (Clinical Outcomes) |
| 2023 | Non-linear dose response effect of cathodal transcranial direct current stimulation on muscle strength in young healthy adults: a randomized controlled study | Wrong outcome (Clinical Outcomes) |
| 2009 | Polarity-sensitive modulation of cortical neurotransmitters by transcranial stimulation | Wrong outcome (fMRI) |
| 2011 | Effects of transcranial direct current stimulation (tDCS) on human regional cerebral blood flow | Wrong outcome (fMRI) |
| 2012 | Cortical activation changes underlying stimulation-induced behavioural gains in chronic stroke | Wrong outcome (fMRI) |
| 2012 | Dynamic modulation of intrinsic functional connectivity by transcranial direct current stimulation | Wrong outcome (fMRI) |
| 2012 | Modulating cortico-striatal and thalamo-cortical functional connectivity with transcranial direct current stimulation | Wrong outcome (fMRI) |
| 2012 | Reorganizing the intrinsic functional architecture of the human primary motor cortex during rest with non-invasive cortical stimulation | Wrong outcome (fMRI) |
| 2013 | Differential effects of dual and unihemispheric motor cortex stimulation in older adults | Wrong outcome (fMRI) |
| 2013 | Transcranial direct current stimulation (tDCS) of left parietal cortex facilitates gesture processing in healthy subjects | Wrong outcome (fMRI) |
| 2014 | Transcranial direct current stimulation modulates activation and effective connectivity during spatial navigation | Wrong outcome (fMRI) |
| 2014 | tDCS modulates cortical nociceptive processing but has little to no impact on pain perception | Wrong outcome (fMRI) |
| 2014 | Cerebellar-parietal connections underpin phonological storage | Wrong outcome (fMRI) |
| 2014 | Polarity-specific effects of motor transcranial direct current stimulation on fMRI resting state networks | Wrong outcome (fMRI) |
| 2014 | Local and remote effects of transcranial direct current stimulation on the electrical activity of the motor cortical network | Wrong outcome (fMRI) |
| 2014 | The value and cost of complexity in predictive modelling: role of tissue anisotropic conductivity and fibre tracts in neuromodulation | Wrong outcome (fMRI) |
| 2015 | The effect of tissue anisotropy on the radial and tangential components of the electric field in transcranial direct current stimulation | Wrong outcome (fMRI) |
| 2015 | Resting-state fMRI reveals enhanced functional connectivity in spatial navigation networks after transcranial direct current stimulation | Wrong outcome (fMRI) |
| 2015 | Feasibility of using high-definition transcranial direct current stimulation (HD-tDCS) to enhance treatment outcomes in persons with aphasia | Wrong outcome (fMRI) |
| 2015 | Excitability of contralateral and ipsilateral projections of corticobulbar pathways recorded as corticobulbar motor evoked potentials of the cricothyroid muscles | Wrong outcome (fMRI) |
| 2016 | Neural correlates of unihemispheric and bihemispheric motor cortex stimulation in healthy young adults | Wrong outcome (fMRI) |
| 2016 | Polarity-dependent misperception of subjective visual vertical during and after transcranial direct current stimulation (tDCS) | Wrong outcome (fMRI) |
| 2017 | tDCS-Induced Modulation of GABA Levels and Resting-State Functional Connectivity in Older Adults | Wrong outcome (fMRI) |
| 2017 | Multifocal tDCS targeting the resting state motor network increases cortical excitability beyond traditional tDCS targeting unilateral motor cortex | Wrong outcome (fMRI) |
| 2018 | Polarity-specific modulation of pain processing by transcranial direct current stimulation - a blinded longitudinal fMRI study | Wrong outcome (fMRI) |
| 2018 | Polarity-specific modulation of central pain processing by transcranial direct current stimulation | Wrong outcome (fMRI) |
| 2018 | Testing assumptions on prefrontal transcranial direct current stimulation: comparison of electrode montages using multimodal fMRI | Wrong outcome (fMRI) |
| 2018 | Age-dependent effects of brain stimulation on network centrality | Wrong outcome (fMRI) |
| 2018 | Modulating Regional Motor Cortical Excitability with Noninvasive Brain Stimulation Results in Neurochemical Changes in Bilateral Motor Cortices | Wrong outcome (fMRI) |
| 2018 | Single-session tDCS over the dominant hemisphere affects contralateral spectral EEG power, but does not enhance neurofeedback-guided event-related desynchronization of the non-dominant hemisphere's sensorimotor rhythm | Wrong outcome (fMRI) |
| 2019 | Traumatic axonal injury influences the cognitive effect of non-invasive brain stimulation | Wrong outcome (fMRI) |
| 2019 | Cerebellar transcranial direct current stimulation modulates the fMRI signal in the cerebellar nuclei in a simple motor task | Wrong outcome (fMRI) |
| 2019 | Increased interhemispheric synchrony underlying the improved athletic performance of rowing athletes by transcranial direct current stimulation | Wrong outcome (fMRI) |
| 2019 | Non-invasive motor cortex neuromodulation reduces secondary hyperalgesia and enhances activation of the descending pain modulatory network | Wrong outcome (fMRI) |
| 2019 | Towards precise brain stimulation: Is electric field simulation related to neuromodulation? | Wrong outcome (fMRI) |
| 2020 | Transcranial Direct Current Stimulation Applied to the Dorsolateral and Ventromedial Prefrontal Cortices in Smokers Modifies Cognitive Circuits Implicated in the Nicotine Withdrawal Syndrome | Wrong outcome (fMRI) |
| 2020 | Effects of transcranial direct current stimulation on GABA and Glx in children: a pilot study | Wrong outcome (fMRI) |
| 2020 | Task load modulates tDCS effects on brain network for phonological processing | Wrong outcome (fMRI) |
| 2021 | Multifocal Transcranial Direct Current Stimulation Modulates Resting-State Functional Connectivity in Older Adults Depending on the Induced Current Density | Wrong outcome (fMRI) |
| 2021 | Extended fmri-guided anodal and cathodal transcranial direct current stimulation targeting perilesional areas in post-stroke aphasia: a pilot randomized clinical trial | Wrong outcome (fMRI) |
| 2021 | In-vivo imaging of targeting and modulation of depression-relevant circuitry by transcranial direct current stimulation: a randomized clinical trial | Wrong outcome (fMRI) |
| 2021 | Effects of transcranial direct current stimulation on brain network connectivity and complexity in motor imagery | Wrong outcome (fMRI) |
| 2021 | Differential Behavioral and Neural Effects of Regional Cerebellar tDCS | Wrong outcome (fMRI) |
| 2021 | Electrode montage-dependent intracranial variability in electric fields induced by cerebellar transcranial direct current stimulation | Wrong outcome (fMRI) |
| 2021 | Effects of tDCS dose and electrode montage on regional cerebral blood flow and motor behavior | Wrong outcome (fMRI) |
| 2022 | Modulation of brain networks during MR-compatible transcranial direct current stimulation | Wrong outcome (fMRI) |
| 2022 | Frontopolar tDCS Induces Frequency-Dependent Changes of Spontaneous Low-Frequency Fluctuations: a Resting-State fMRI Study | Wrong outcome (fMRI) |
| 2022 | tDCS modulates effective connectivity during motor command following a potential therapeutic target for disorders of consciousness | Wrong outcome (fMRI) |
| 2023 | Robust enhancement of motor sequence learning with 4 mA transcranial electric stimulation | Wrong outcome (fMRI) |
| 2023 | Anodal cerebellar stimulation increases cortical activation: Evidence for cerebellar scaffolding of cortical processing | Wrong outcome (fMRI) |
| 2023 | Modulating Cortical Hemodynamic Activity in Parkinson's Disease Using Focal Transcranial Direct Current Stimulation: A Pilot Functional Near-infrared Spectroscopy Study | Wrong outcome (fMRI) |
| 2017 | Modulation of physiologic artifacts during concurrent tDCS and EEG | Wrong outcomes (EEG) |
| 2018 | Modulation of cortical responses by transcranial direct current stimulation of dorsolateral prefrontal cortex: a resting-state EEG and TMS-EEG study | Wrong outcomes (EEG) |
| 2018 | Cognitive Enhancement Induced by Anodal tDCS Drives Circuit-Specific Cortical Plasticity | Wrong outcomes (EEG) |
| 2019 | Cortico-Muscular Coherence Modulated by High-Definition Transcranial Direct Current Stimulation in People With Chronic Stroke | Wrong outcomes (EEG) |
| 2020 | Targeting the anterior cingulate with bipolar and high-definition transcranial direct current stimulation | Wrong outcomes (EEG) |
| 2021 | Revisiting the effects of transcranial direct current stimulation on pattern-reversal visual evoked potentials | Wrong outcomes (EEG) |
| 2022 | No effects of transcranial direct current stimulation on visual evoked potential and peak gamma frequency | Wrong outcomes (EEG) |
| 2012 | Abnormal changes of synaptic excitability in migraine with aura | Wrong patient population |
| 2018 | Revisiting interhemispheric imbalance in chronic stroke: a tDCS study | Wrong patient population |
| 2021 | Effects of Transcranial Direct Current Stimulation and High-Definition Transcranial Direct Current Stimulation Enhanced Motor Learning on Robotic Transcranial Magnetic Stimulation Motor Maps in Children | Wrong patient population |
| 2019 | Safety and feasibility of transcranial direct current stimulation in amyotrophic lateral sclerosis - a pilot study with a single subject experimental design | Wrong study design |
| 2013 | Induction of cortical plasticity and improved motor performance following unilateral and bilateral transcranial direct current stimulation of the primary motor cortex. | Wrong study design |
| 2018 | Effects of Cathode Location and the Size of Anode on Anodal Transcranial Direct Current Stimulation Over the Leg Motor Area in Healthy Humans. | Wrong study design |

**Appendix G. Risk of Bias Assessment**

| **Study** | **1** | **2** | **3** | **4** | **5** | **6** | **7** | **8** | **9** | **10** | **11** | **12** | **13** | **Quality** |
| --- | --- | --- | --- | --- | --- | --- | --- | --- | --- | --- | --- | --- | --- | --- |
| Agboada. 2019 | Y | Y | Y | Y | N | Y | N | Y | Y | Y | Y | Y | Y | Low |
| Alonzo. 2011 | Y | Y | Y | N | N | Y | N | Y | Y | Y | Y | Y | Y | Low |
| Ammann. 2017 | Y | Y | Y | Y | N | Y | N | Y | Y | Y | Y | Y | Y | Low |
| Anguis. 2018 | Y | Y | Y | Y | Y | Y | N | Y | N | Y | Y | Y | Y | Low |
| Azabou. 2013 | Y | Y | Y | N | N | Y | N | Y | N | Y | Y | Y | N | Low |
| Baltar. 2018 | Y | Y | Y | Y | N | Y | Y | Y | Y | Y | Y | Y | Y | Low |
| Bashir. 2019 | Y | Y | Y | Y | N | Y | N | Y | Y | Y | Y | Y | Y | Low |
| Bashir. 2021 | Y | Y | Y | N | N | Y | N | Y | Y | Y | Y | N | Y | Low |
| Bastani. 2014 | Y | Y | Y | Y | N | Y | N | Y | Y | Y | Y | Y | Y | Low |
| Bastani. 2013 | Y | Y | Y | Y | N | Y | N | Y | Y | Y | Y | Y | Y | Low |
| Batsikadze. 2013 | Y | Y | Y | Y | N | Y | N | Y | Y | Y | Y | Y | Y | Low |
| Behrangrad. 2022 | Y | Y | Y | Y | N | Y | Y | Y | Y | Y | Y | Y | Y | Low |
| Bocci. 2015 | Y | Y | Y | Y | N | Y | Y | Y | Y | Y | Y | N | Y | Low |
| Boros. 2008 | Y | Y | Y | Y | N | Y | N | Y | Y | Y | Y | Y | Y | Low |
| Cabibel. 2018 | Y | Y | Y | N | N | Y | N | Y | Y | Y | Y | Y | Y | Low |
| Chew. 2015 | Y | Y | Y | Y | N | Y | Y | Y | Y | Y | Y | Y | Y | Low |
| Christova. 2015 | Y | Y | Y | Y | N | Y | N | Y | N | Y | Y | Y | Y | Low |
| Cosentino. 2017 | Y | Y | Y | Y | Y | Y | N | Y | Y | Y | Y | Y | Y | Low |
| Faber. 2017 | Y | Y | Y | Y | Y | Y | N | Y | Y | Y | Y | Y | Y | Low |
| Farnad. 2021 | Y | Y | Y | Y | N | Y | N | Y | Y | Y | Y | Y | Y | Low |
| Feurra. 2011 | Y | Y | Y | N | N | Y | N | Y | Y | Y | Y | Y | Y | Low |
| Foerster. 2019 | Y | Y | Y | Y | N | Y | N | Y | Y | Y | Y | N | Y | Low |
| Furubayashi. 2008 | Y | Y | Y | N | N | Y | N | Y | Y | Y | Y | Y | Y | Low |
| Galea. 2009 | Y | Y | Y | Y | N | Y | Y | Y | Y | Y | Y | Y | Y | Low |
| Galvez. 2013 | Y | Y | Y | Y | N | Y | N | Y | Y | Y | Y | Y | Y | Low |
| Ghasemian-Shirvan. 2020 | Y | Y | Y | Y | N | Y | N | Y | Y | Y | Y | Y | Y | Low |
| Haeckert. 2020 | Y | Y | Y | Y | N | Y | N | Y | Y | Y | Y | Y | Y | Low |
| Hashemirad. 2017 | Y | Y | Y | Y | N | Y | N | Y | Y | Y | Y | Y | Y | Low |
| Hassanzahraee. 2020 | Y | Y | Y | Y | Y | Y | Y | Y | Y | Y | Y | Y | Y | High |
| Hill. 2017 | Y | Y | Y | Y | N | Y | N | Y | Y | Y | Y | Y | Y | Low |
| Horvath. 2016 | Y | Y | Y | Y | N | Y | N | Y | Y | Y | Y | Y | Y | Low |
| Isis. 2023 | Y | Y | Y | Y | N | Y | Y | Y | Y | Y | Y | Y | Y | Low |
| Jamil. 2019 | Y | Y | N | Y | N | Y | N | Y | Y | N | Y | Y | Y | Low |
| Jefferson. 2009 | Y | Y | Y | Y | N | Y | Y | Y | Y | Y | Y | N | Y | Low |
| Jeffrey. 2007 | Y | Y | Y | Y | N | Y | N | Y | Y | Y | Y | Y | Y | Low |
| Khedr. 2013 | Y | Y | Y | Y | Y | Y | N | Y | N | Y | Y | Y | Y | Low |
| Kidgell. 2013 | Y | Y | Y | Y | N | Y | Y | Y | Y | Y | Y | Y | Y | Low |
| Kindred. 2019 | Y | Y | Y | Y | Y | Y | N | Y | Y | Y | Y | Y | Y | Low |
| Kuo. 2013 | Y | Y | Y | N | N | Y | N | Y | Y | Y | Y | Y | Y | Low |
| Kuo. 2020 | Y | Y | Y | N | N | Y | N | Y | Y | Y | Y | Y | Y | Low |
| Laczo. 2014 | Y | Y | Y | Y | N | Y | N | Y | Y | Y | Y | Y | Y | Low |
| Lampropoulou. 2013 | Y | Y | Y | Y | Y | Y | N | Y | Y | Y | Y | Y | Y | Low |
| Lefebvre. 2019 | Y | Y | N | Y | Y | Y | N | Y | Y | Y | Y | Y | Y | Low |
| McCambridge. 2015 | Y | Y | Y | Y | Y | Y | Y | Y | Y | Y | Y | Y | Y | High |
| Moliadze. 2010 | N | Y | Y | N | N | Y | N | Y | Y | Y | Y | N | Y | Low |
| Monte-Silva. 2010 | N | Y | Y | N | N | Y | N | Y | Y | Y | Y | Y | Y | Low |
| Monte-Silva. 2013 | Y | Y | Y | Y | Y | Y | N | Y | Y | Y | Y | Y | Y | Low |
| Mooney. 2018 | Y | Y | Y | Y | Y | Y | N | Y | Y | Y | Y | Y | Y | Low |
| Mordillo-Mateos. 2012 | Y | Y | Y | N | N | Y | N | Y | Y | Y | Y | Y | Y | Low |
| Mosayebi-Samani. 2023 | Y | Y | Y | Y | N | Y | N | Y | Y | Y | Y | Y | Y | Low |
| Murray. 2015 | Y | Y | Y | Y | N | Y | N | Y | Y | Y | Y | Y | Y | Low |
| NCT03481309 | Y | Y | Y | Y | Y | Y | Y | Y | N | Y | Y | N | Y | Low |
| Nitsche. 2007 | Y | Y | N | N | N | Y | N | Y | Y | Y | Y | Y | Y | Low |
| Nitsche. 2003 | Y | Y | Y | N | N | Y | N | Y | Y | Y | Y | Y | Y | Low |
| O'Shea. 2014 | N | Y | Y | Y | N | Y | N | Y | Y | Y | Y | Y | Y | Low |
| Pellerini. 2021 | Y | Y | Y | N | N | Y | N | Y | Y | Y | Y | Y | Y | Low |
| Pillen. 2022 | Y | Y | Y | N | N | Y | N | Y | Y | Y | Y | Y | Y | Low |
| Power. 2006 | Y | Y | Y | Y | N | Y | N | Y | Y | Y | Y | Y | Y | Low |
| Puri. 2016 | N | Y | Y | N | N | Y | N | Y | Y | Y | Y | Y | Y | Low |
| Rivera-Urbina. 2015 | Y | Y | Y | Y | N | Y | N | Y | Y | Y | Y | Y | Y | Low |
| Samani. 2019 | Y | Y | Y | Y | N | Y | N | Y | Y | Y | Y | Y | Y | Low |
| Santarnecchi. 2014 | Y | Y | Y | Y | N | Y | N | Y | Y | Y | Y | Y | Y | Low |
| Sasaki. 2016 | Y | Y | Y | Y | N | Y | N | Y | Y | Y | Y | Y | Y | Low |
| Schabrun. 2013 | Y | Y | Y | Y | N | Y | N | Y | Y | Y | Y | Y | Y | Low |
| Shah. 2013 | Y | Y | Y | Y | N | Y | Y | Y | Y | Y | Y | Y | Y | Low |
| Sohn. 2012 | Y | Y | Y | Y | N | Y | Y | Y | N | Y | Y | Y | Y | Low |
| Strube. 2016 | Y | Y | Y | N | N | Y | N | Y | Y | Y | Y | Y | Y | Low |
| Suzuki. 2012 | Y | Y | Y | N | N | Y | N | Y | Y | Y | Y | Y | Y | Low |
| Thapa. 2018 | Y | Y | Y | N | N | Y | N | Y | Y | Y | Y | Y | Y | Low |
| Tremblay. 2016 | Y | Y | Y | Y | N | Y | N | Y | Y | Y | Y | N | Y | Low |
| Uehara. 2023 | Y | Y | Y | Y | N | Y | Y | Y | Y | Y | Y | Y | Y | Low |
| Vaseghi. 2016 | Y | Y | Y | Y | N | Y | N | Y | Y | Y | Y | Y | Y | Low |
| Vaseghi. 2015 | Y | Y | Y | Y | N | Y | N | Y | Y | Y | Y | Y | Y | Low |
| Vaseghi. 2015 | Y | Y | Y | Y | N | Y | Y | Y | Y | Y | Y | Y | Y | Low |
| Vignaud. 2018 | Y | Y | Y | N | N | Y | N | Y | Y | Y | Y | Y | Y | Low |
| Vitale. 2021 | Y | Y | Y | Y | N | Y | N | Y | Y | Y | Y | Y | Y | Low |
| Wiethoff. 2014 | Y | Y | Y | Y | N | Y | Y | Y | Y | Y | Y | Y | Y | Low |
| Wiltshire. 2020 | Y | Y | Y | Y | Y | Y | Y | Y | Y | Y | Y | Y | Y | High |
| Wong. 2022 | Y | Y | Y | Y | N | Y | Y | Y | Y | Y | Y | Y | Y | Low |
| Wong. 2022 | Y | Y | Y | Y | N | Y | Y | Y | Y | Y | Y | Y | Y | Low |

**Appendix H. Model output evaluating the effects of transcranial direct current stimulation over time: Polarity**


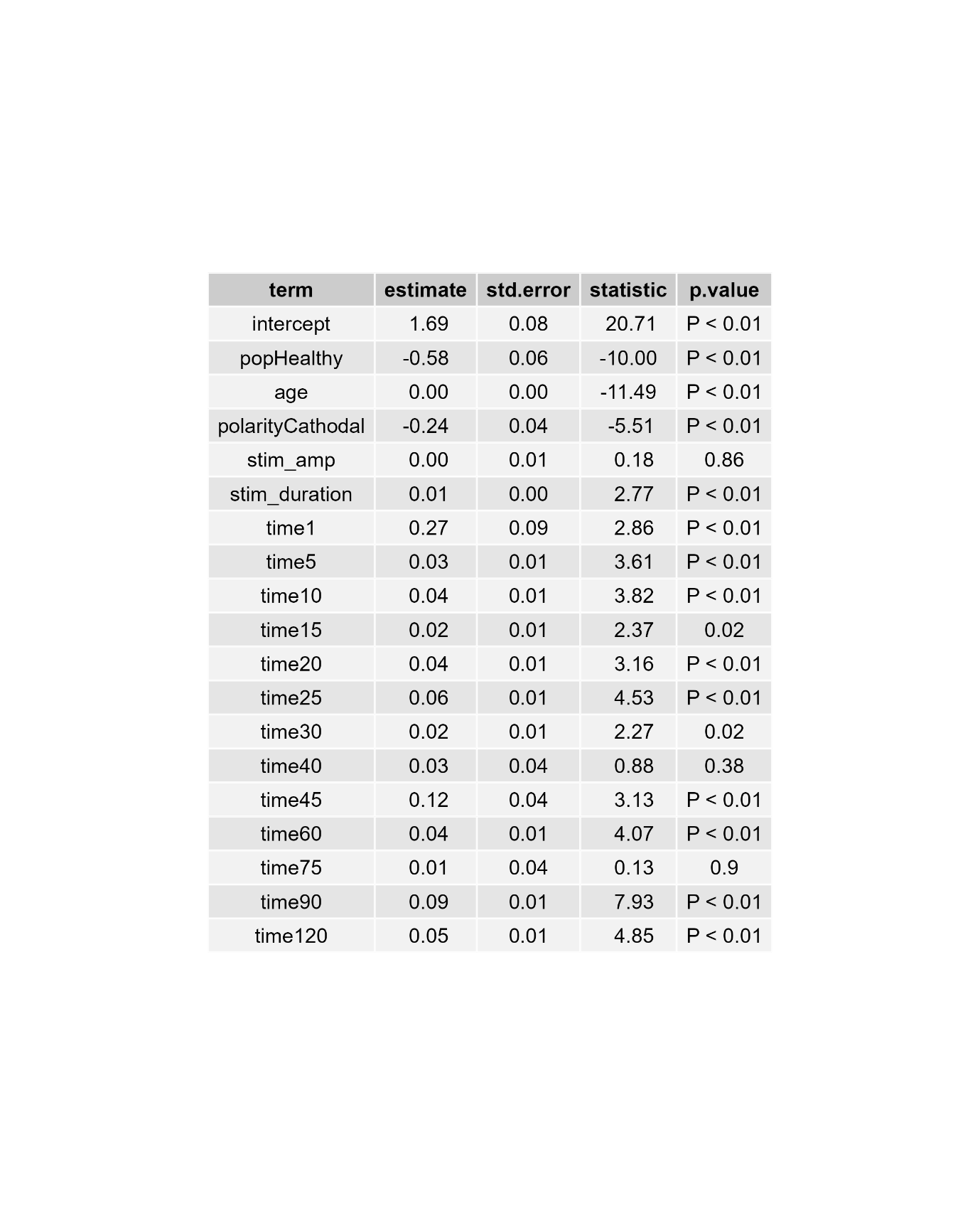


**Appendix I. Individual study level effects of transcranial direct current stimulation over time: Polarity**


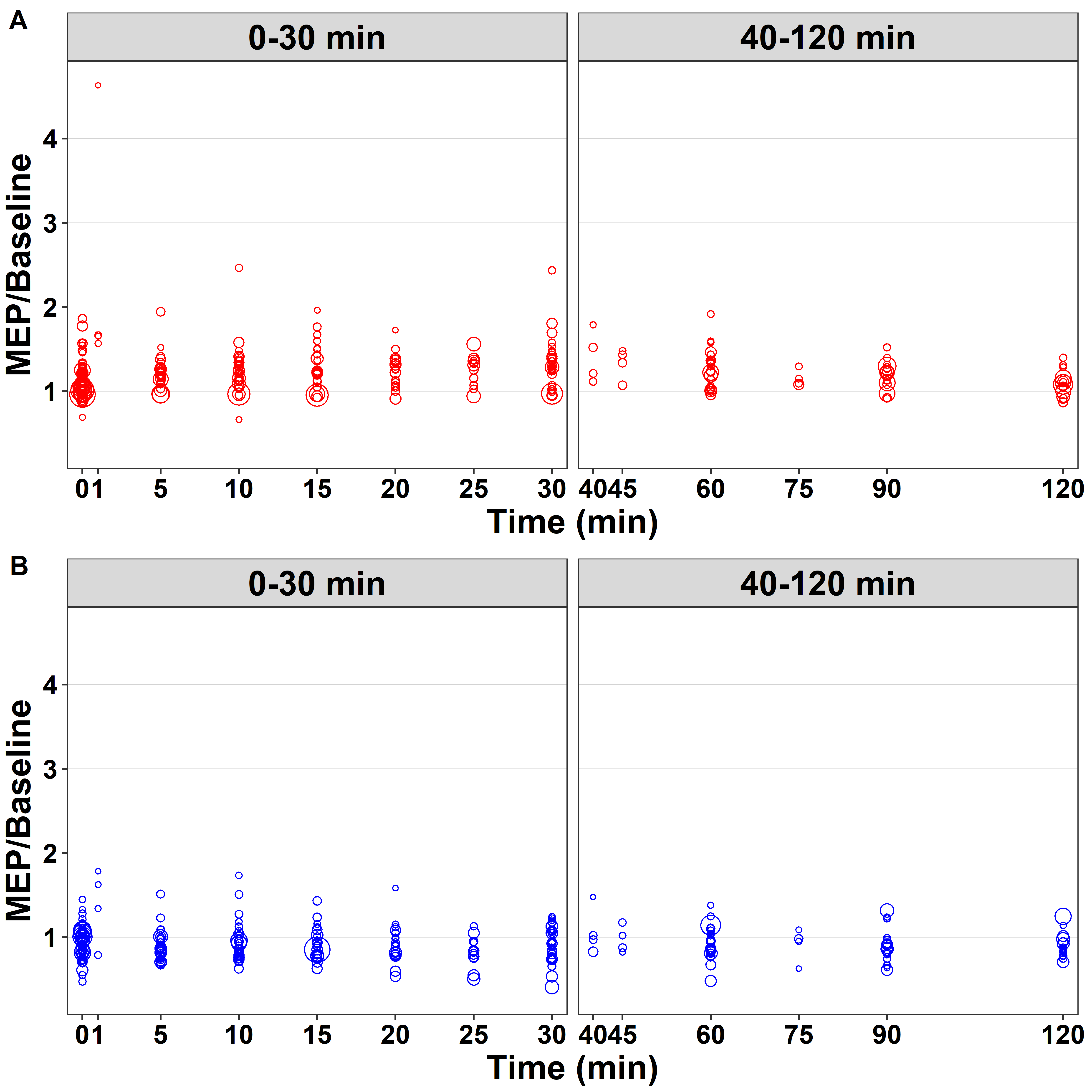


**Appendix J. Model output evaluating the effects of anodal transcranial direct current stimulation over time: Stimulation Duration**


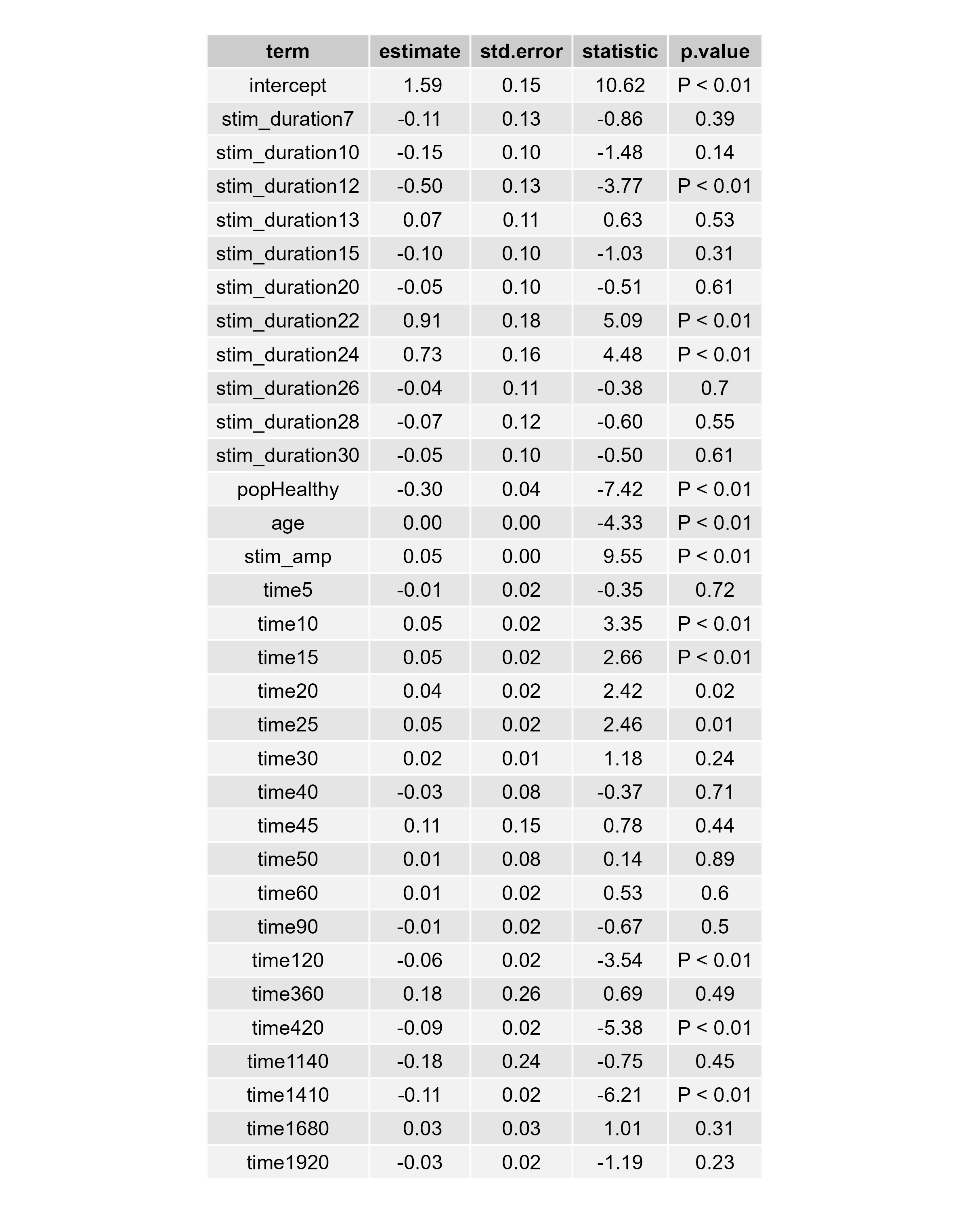


**Appendix K. Predicted values of motor evoked potential-to-baseline for stimulation duration: A) Anodal tDCS, B) Cathodal tDCS**

**
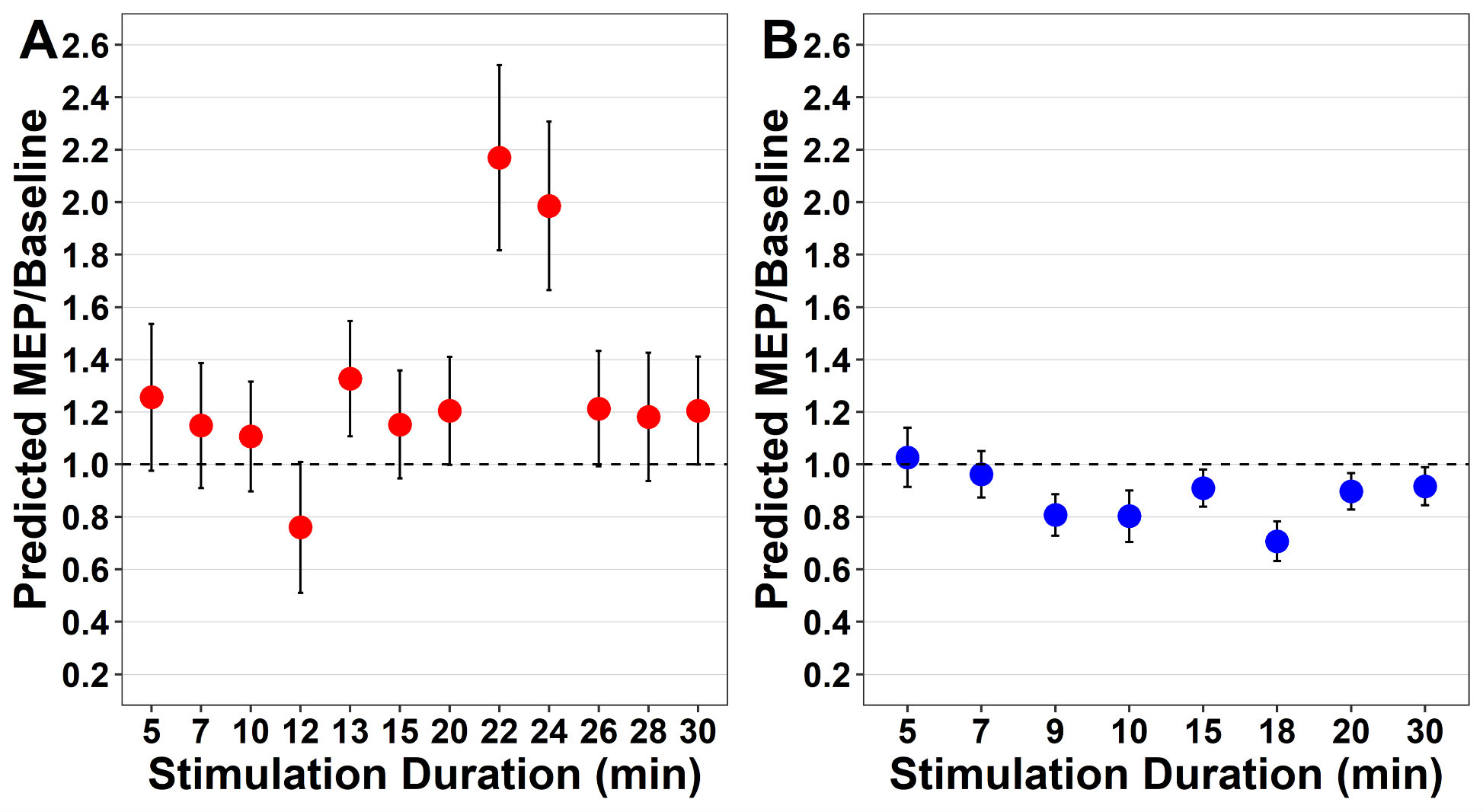
**

**Appendix L. Model output evaluating the effects of cathodal transcranial direct current stimulation over time: Stimulation Duration**


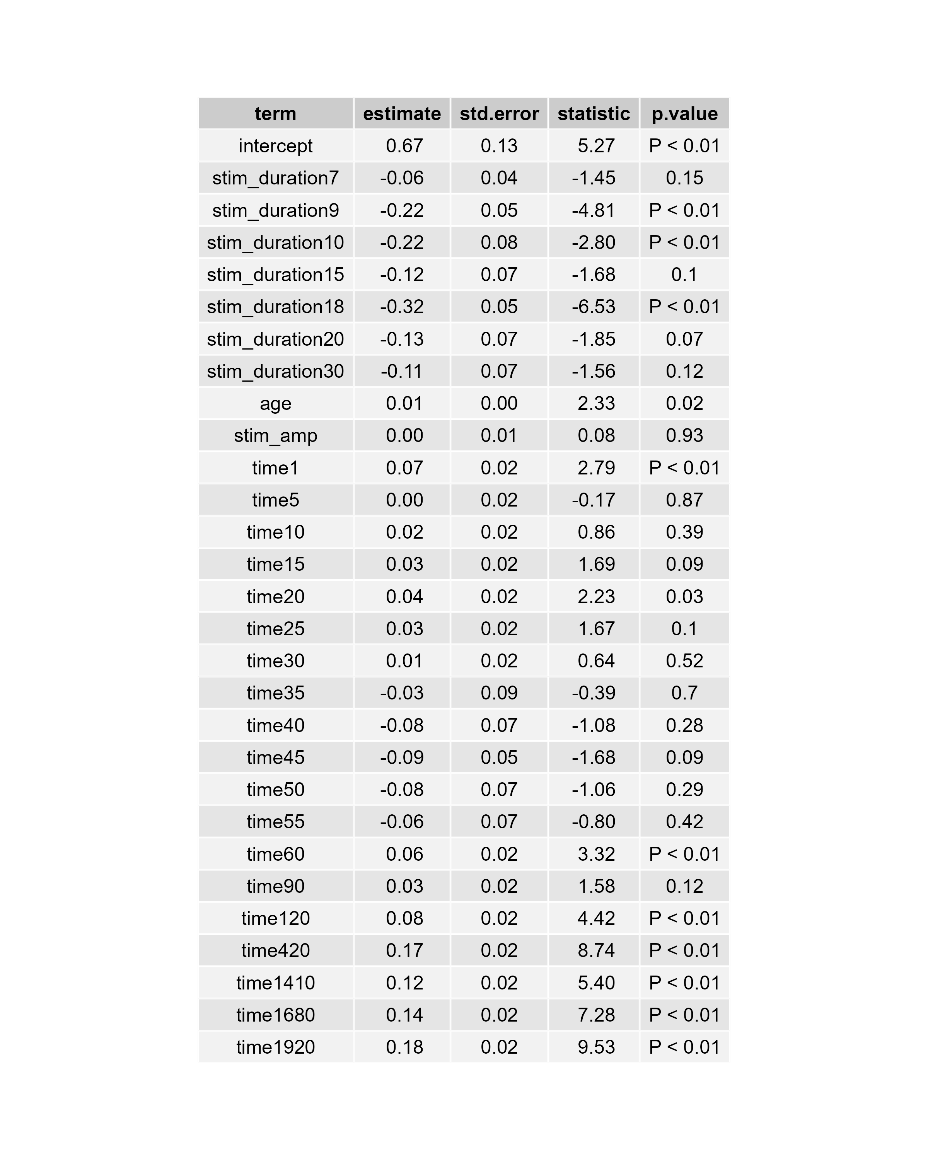


**Appendix M. Model output evaluating the effects of anodal transcranial direct current stimulation over time: Stimulation Intensity**


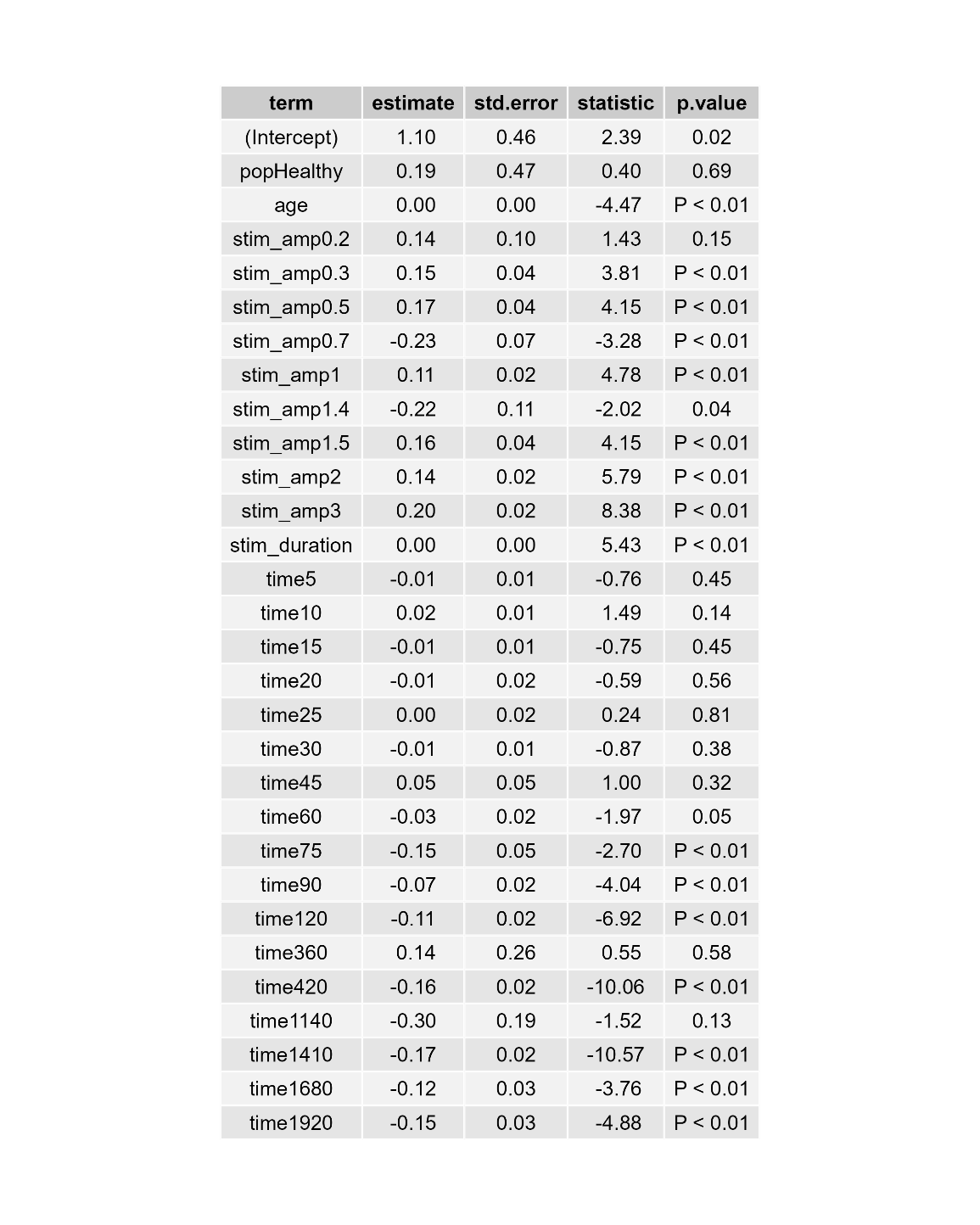


**Appendix N. Predicted values of motor evoked potential-to-baseline for stimulation intensity: A) Anodal tDCS, B) Cathodal tDCS**

**
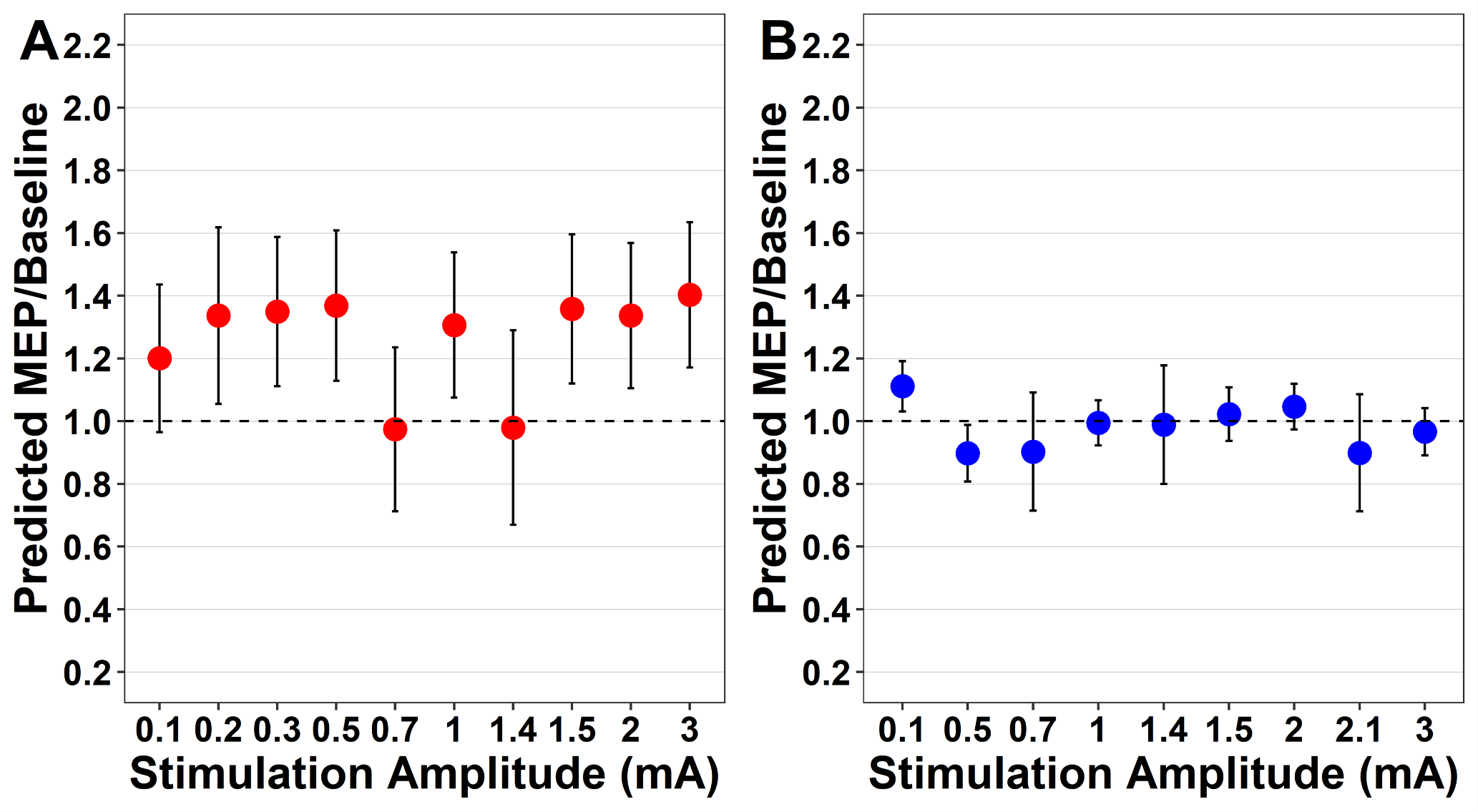
**

**Appendix O. Model output evaluating the effects of cathodal transcranial direct current stimulation over time: Stimulation Intensity**


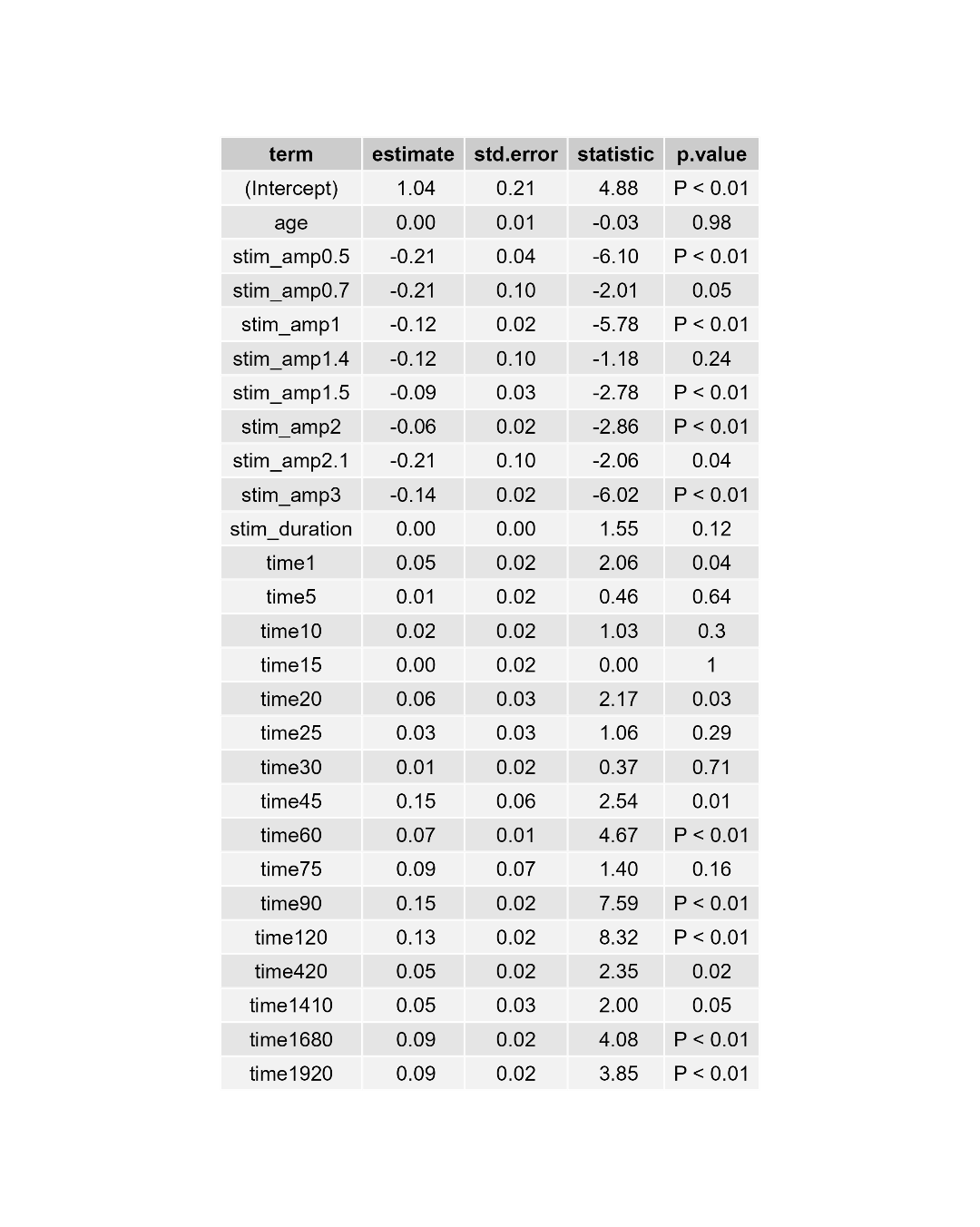


**Appendix P. Model output evaluating the effects of anodal transcranial direct current stimulation over time: Stimulation Frequency**


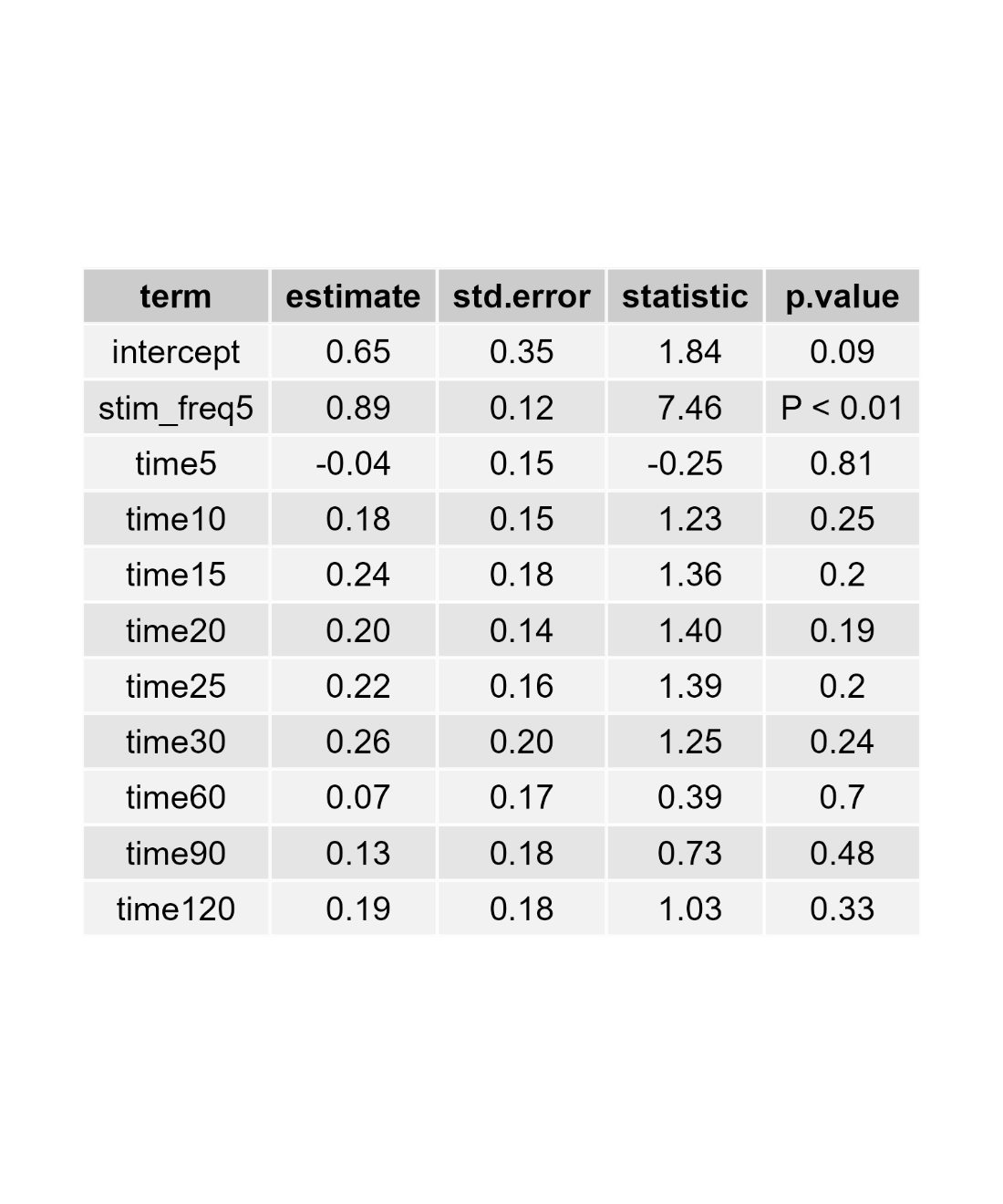


**Appendix Q. Predicted values of motor evoked potential-to-baseline for anodal stimulation frequency**

**
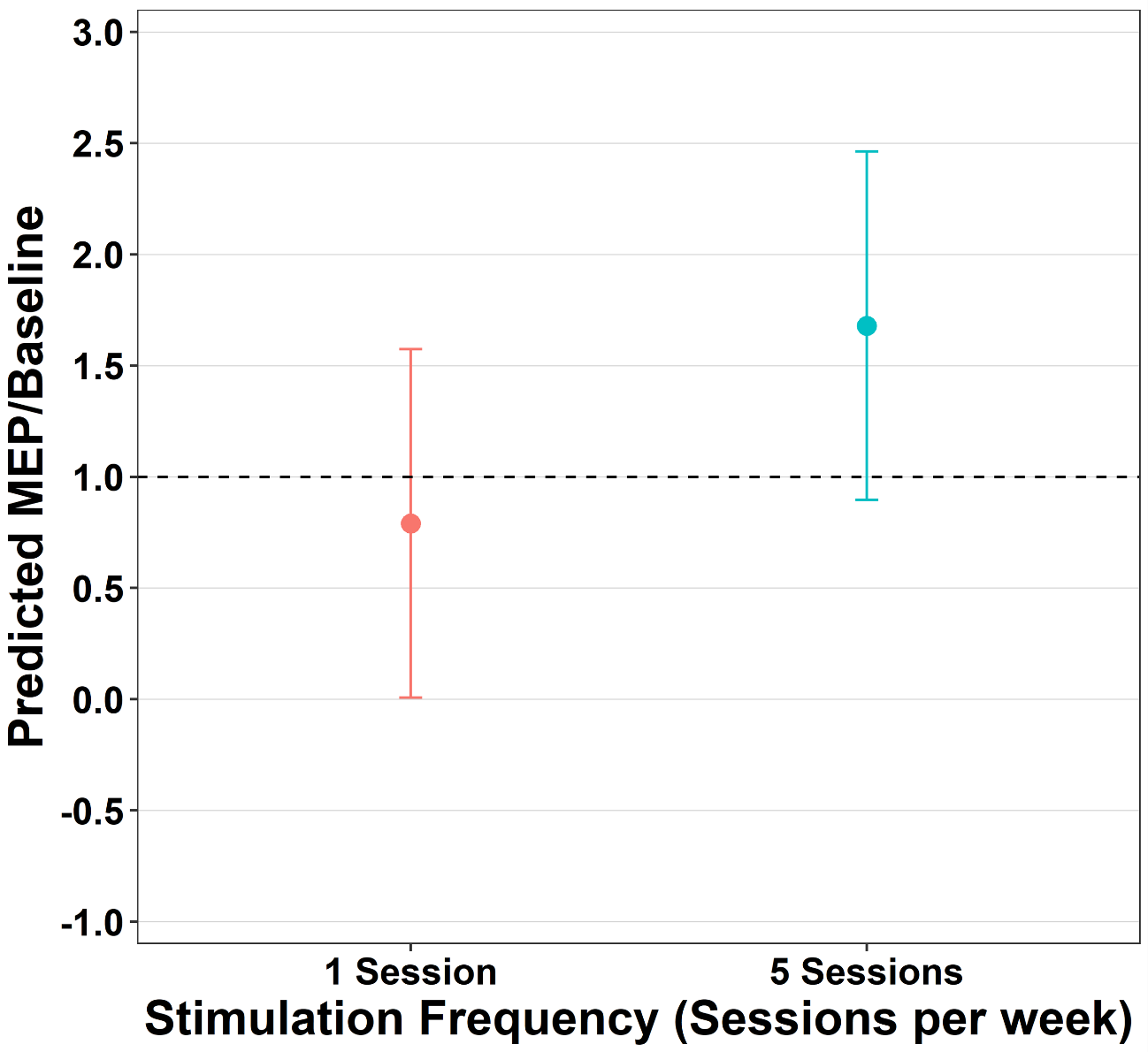
**

**Appendix R. Model output evaluating the effects of anodal transcranial direct current stimulation over time: Electrode Montage**


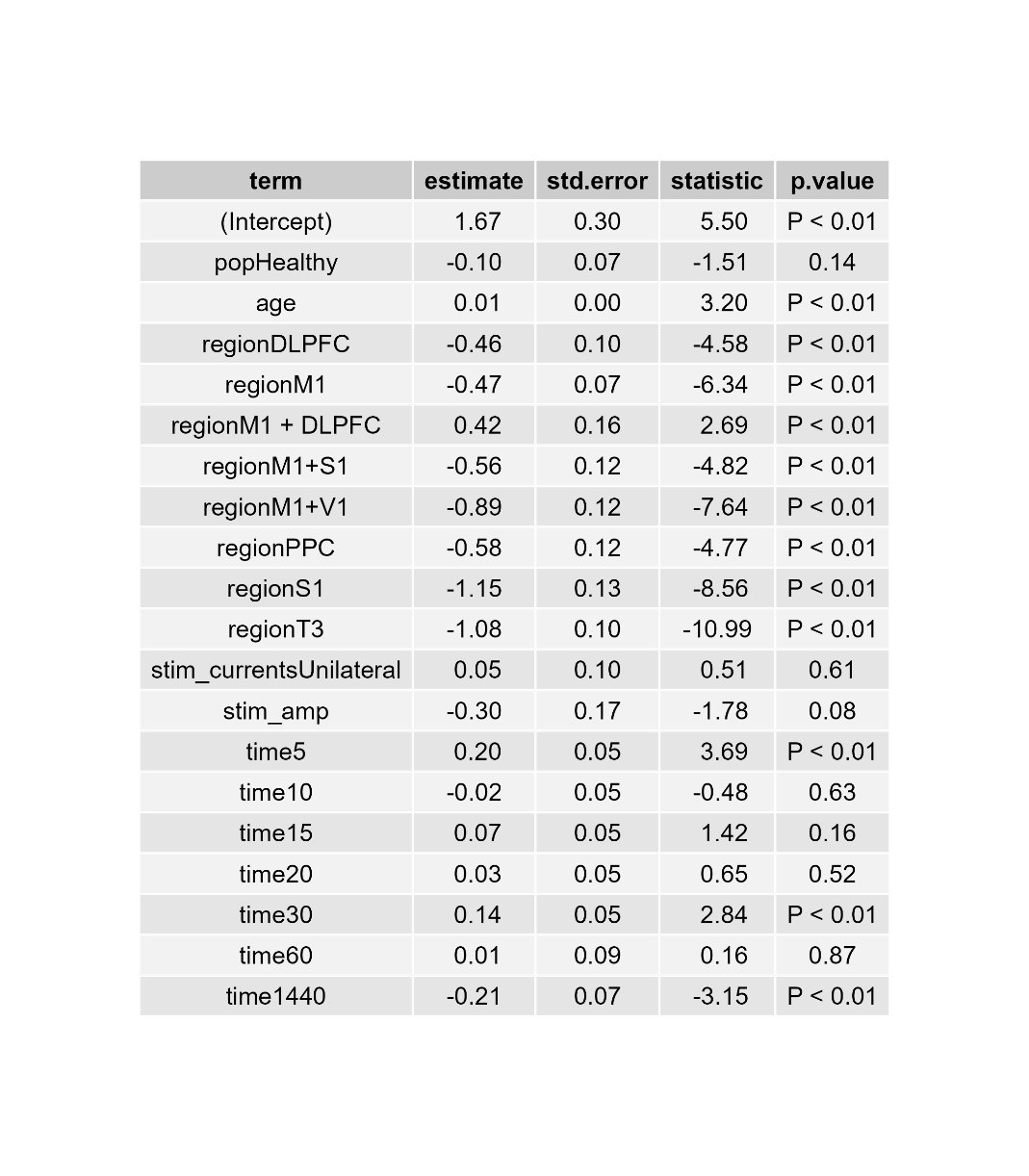


**Appendix S. Predicted values of motor evoked potential-to-baseline for electrode montage: A) Anodal tDCS, B) Cathodal tDCS**

**
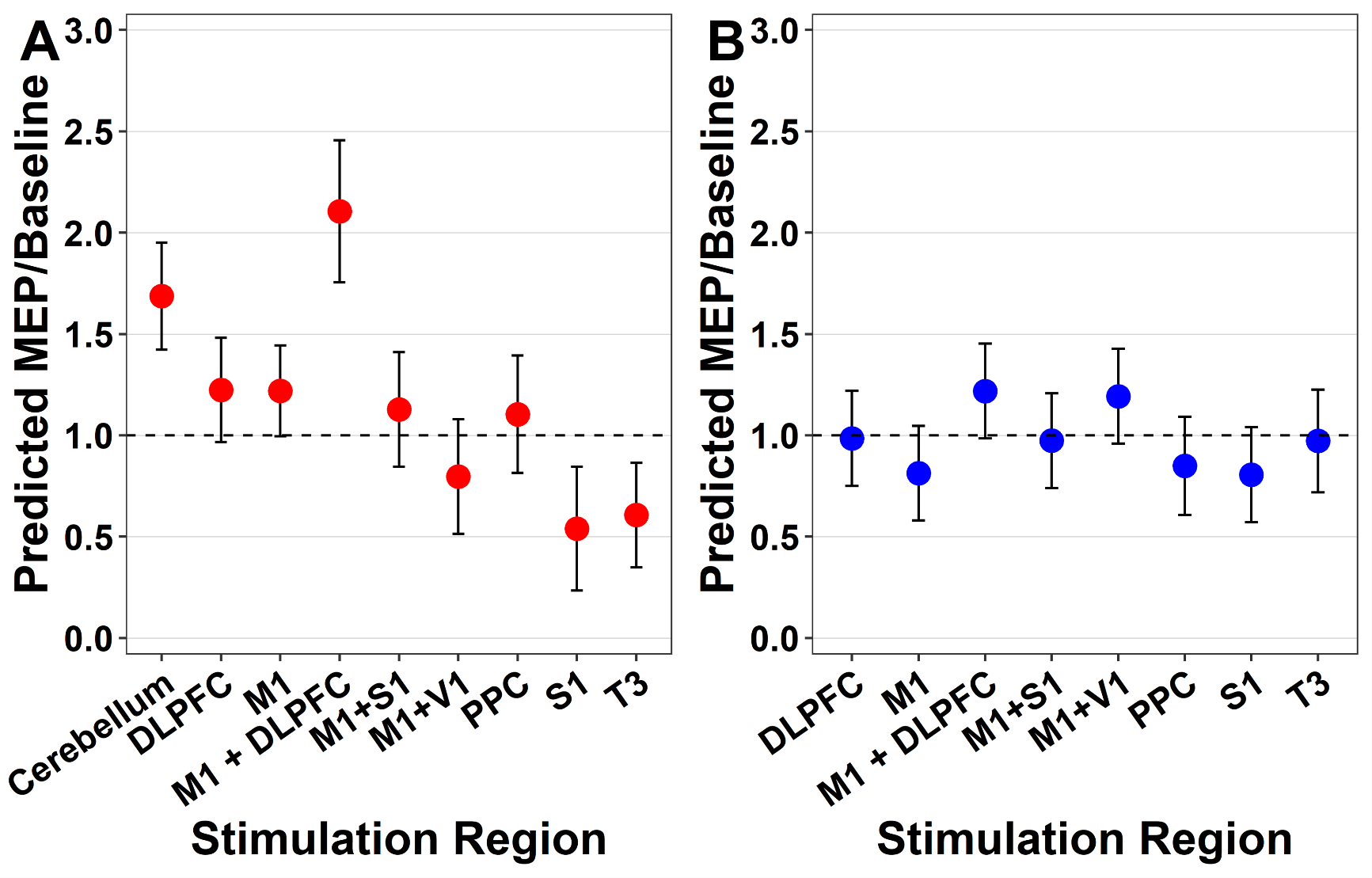
**


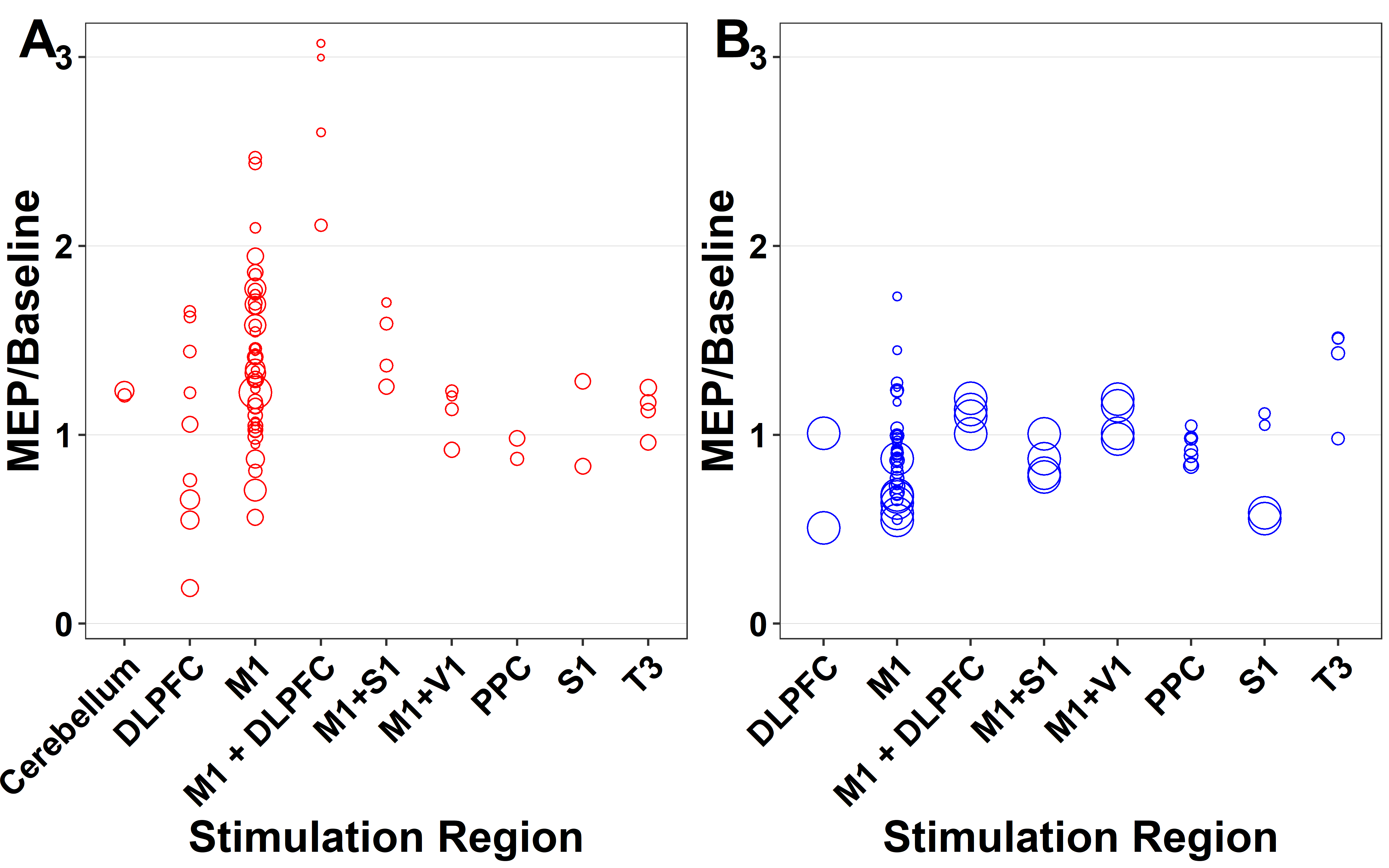


**Appendix T. Model output evaluating the effects of cathodal transcranial direct current stimulation over time: Electrode Montage**


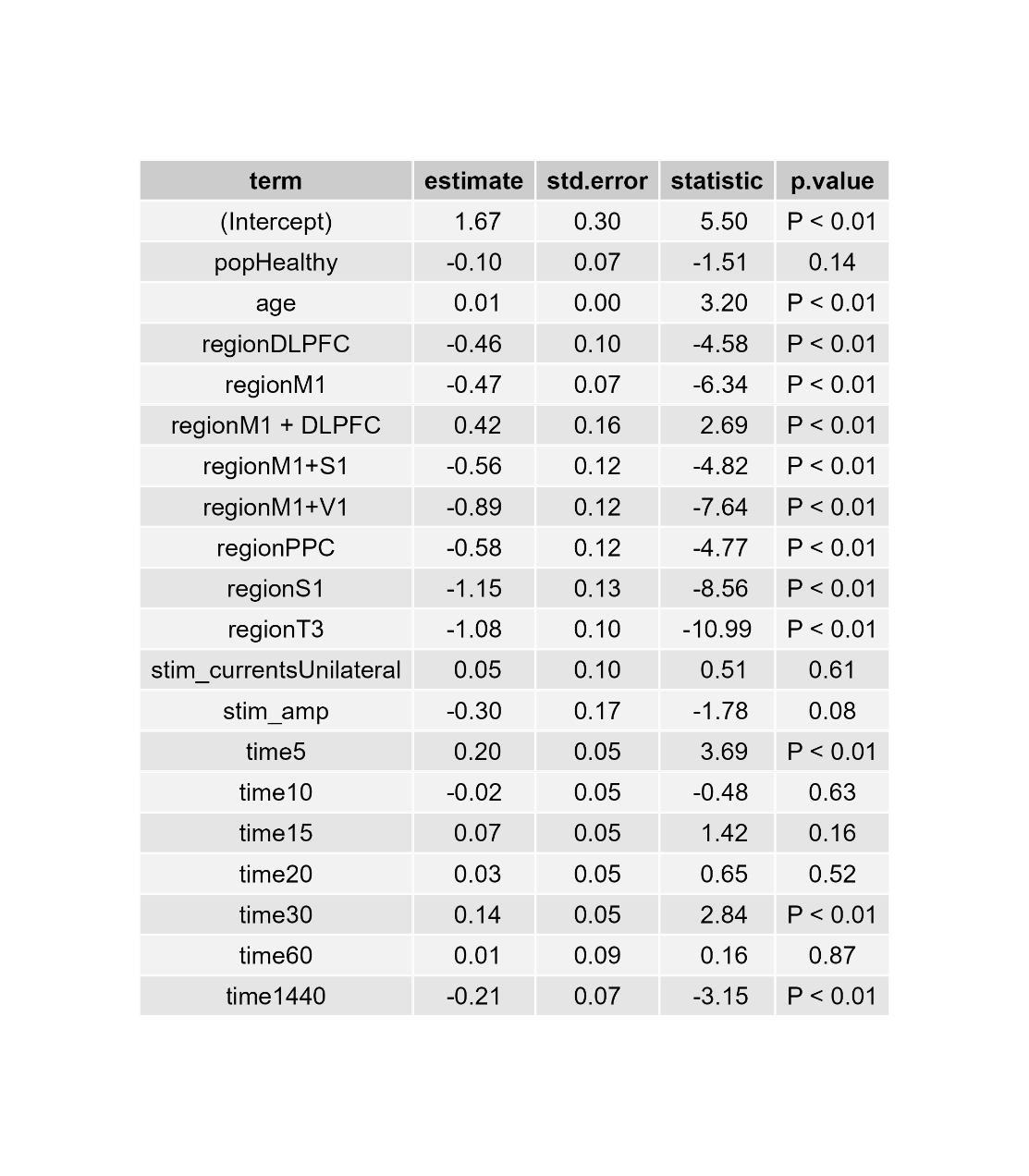


**Appendix U. Predicted values of motor evoked potential-to-baseline for electrode design: A) Anodal tDCS, B) Cathodal tDCS**

**
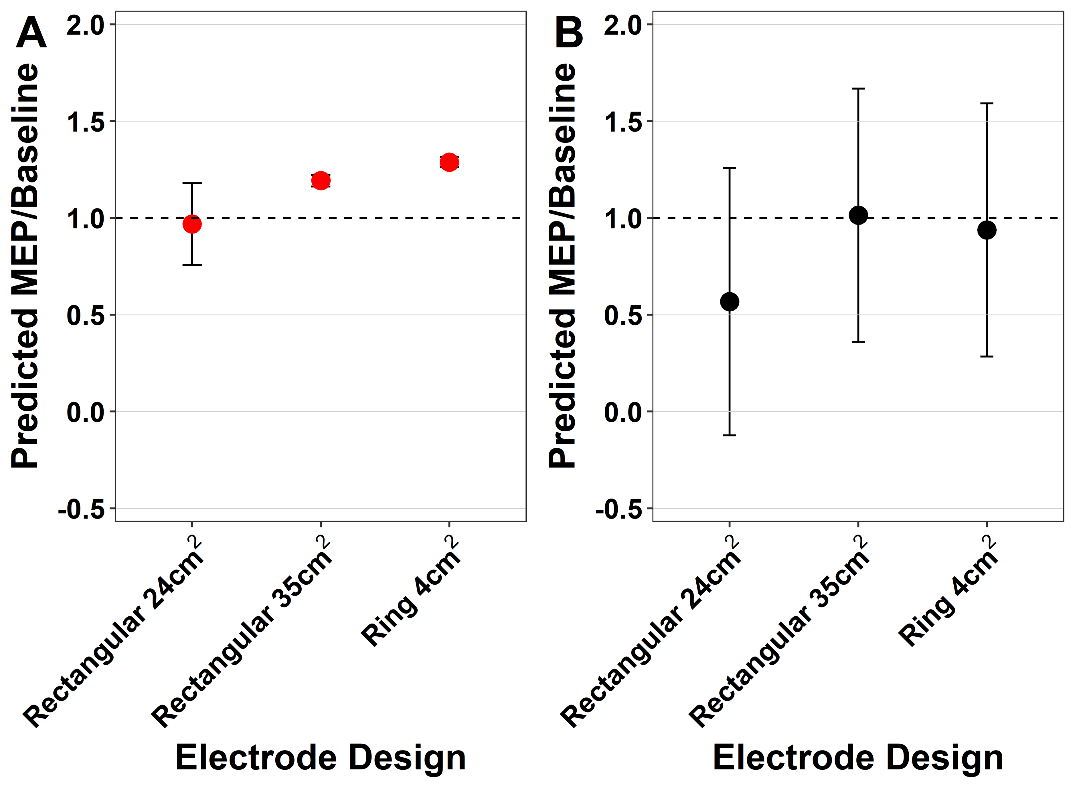
**

**Appendix V. Model output evaluating the effects of anodal transcranial direct current stimulation over time: Electrode Design**


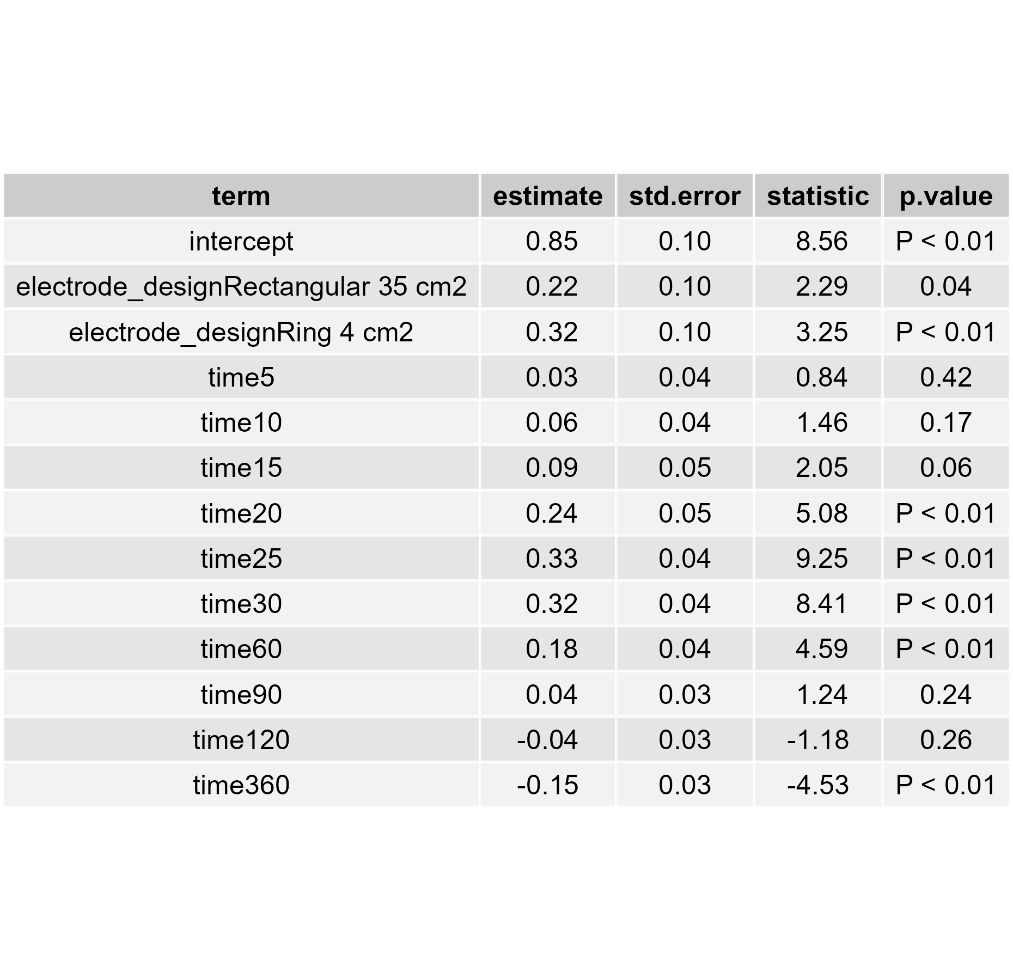


**Appendix W. Individual study level effects of anodal transcranial direct current stimulation over time: Electrode Design**


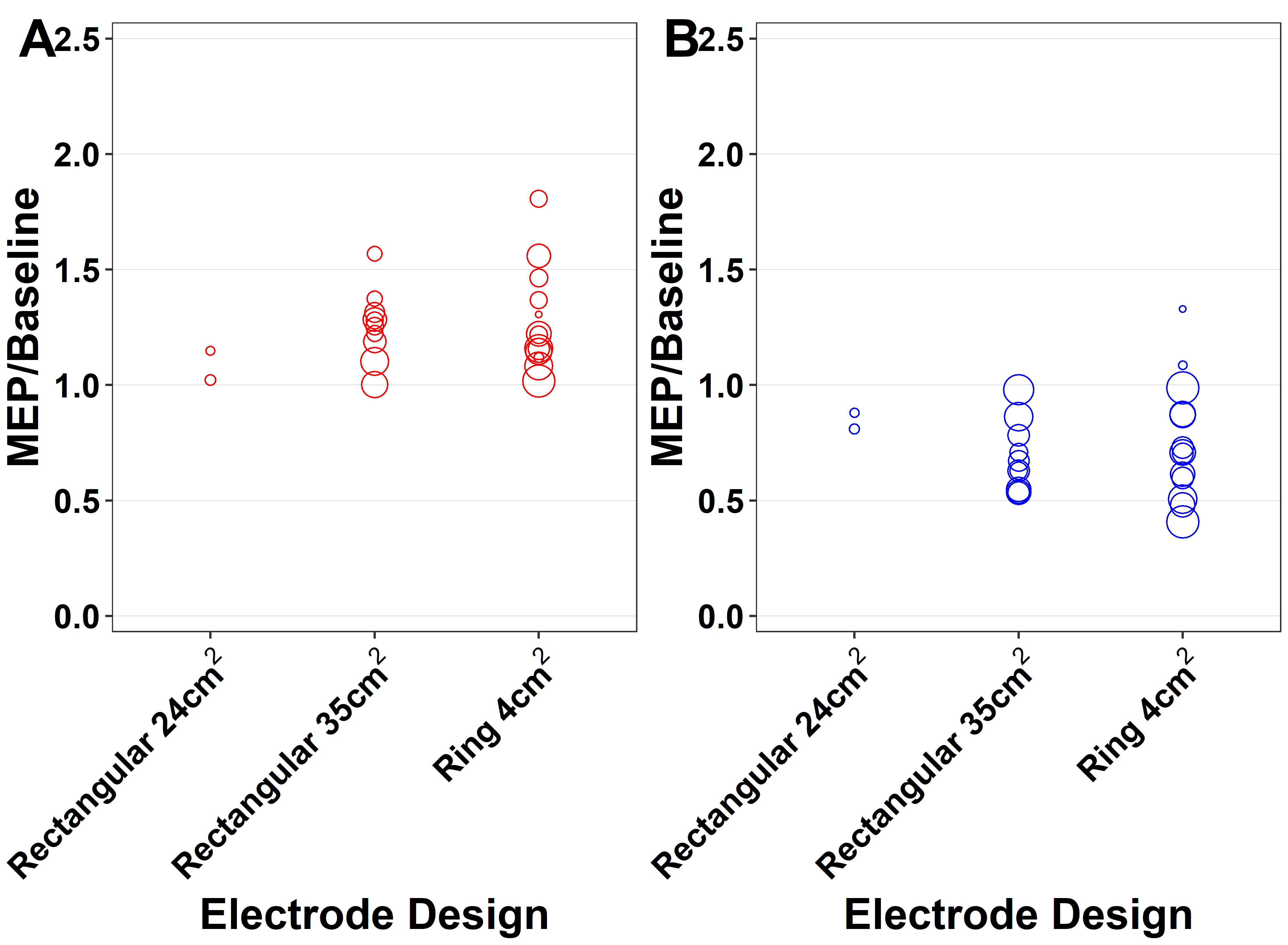


**Appendix X. Model output evaluating the effects of cathodal transcranial direct current stimulation over time: Electrode Montage**


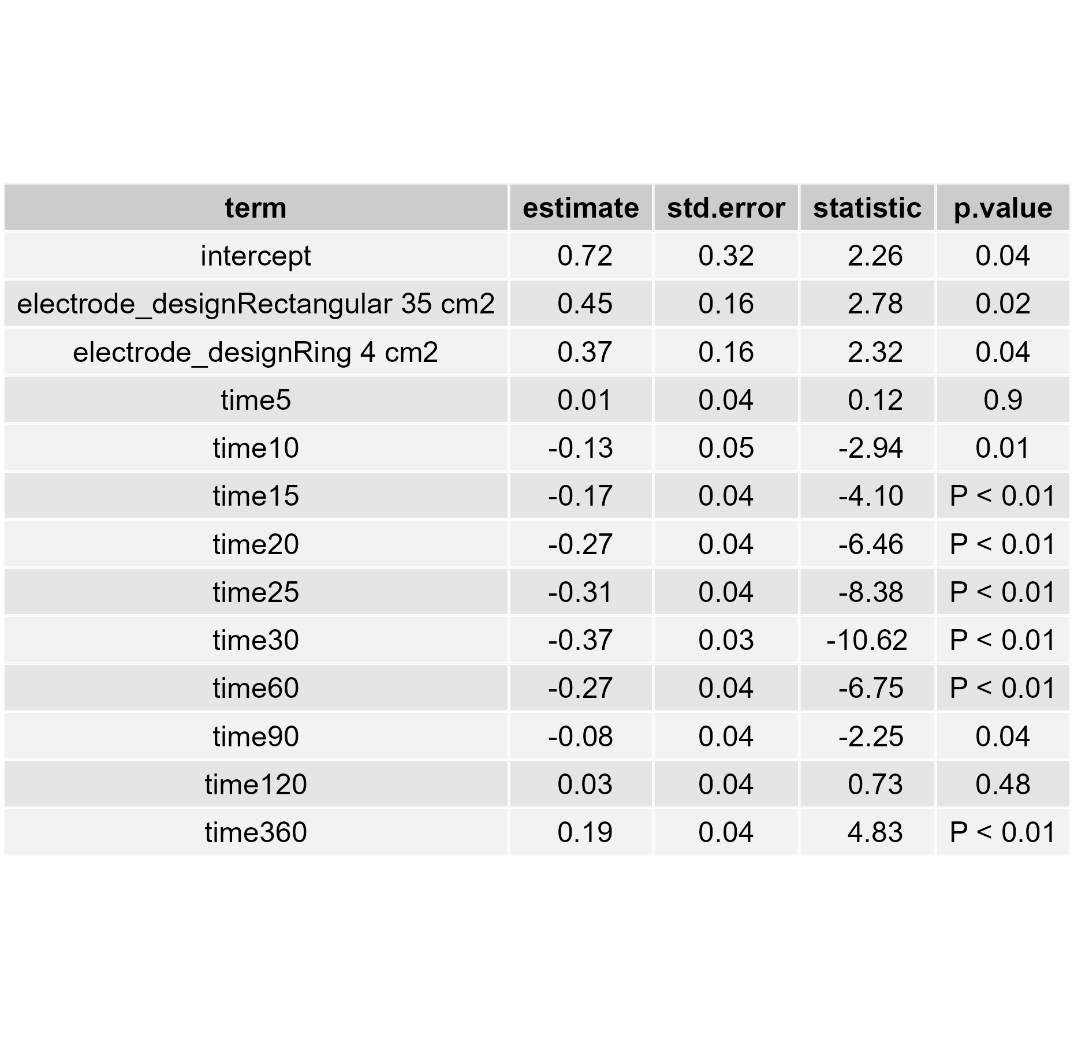

Supplement: Supplementary file 1 — Additional file1 (DOCX 2529 KB) [file 42466_2025_449_MOESM1_ESM.docx]
